# Factors Associated With Social and Emotional Wellbeing Among Aboriginal and Torres Strait Islander LGBTQA+ Young People

**DOI:** 10.5694/mja2.70333

**Published:** 2026-11-11

**Authors:** Bep Uink, Shakara Liddelow‐Hunt, Benjamin Harrap, Kate Daglas, James H. L. Hill, Lily Hayward, Braden Hill, Yael Perry, Ashleigh Lin

**Affiliations:** ^1^ Australian Indigenous HealthInfoNet Edith Cowan University Mount Lawley Western Australia Australia; ^2^ Australian Research Centre in Sex, Health and Society La Trobe University Melbourne Victoria Australia; ^3^ Youth Mental Health The Kids Research Institute Australia Nedlands Western Australia Australia; ^4^ Yardhura Walani Australian National University Acton Australian Capital Territory Australia; ^5^ Department of Infectious Diseases The University of Melbourne Melbourne Victoria Australia; ^6^ Youth Advisory Group Walkern Katatdjin Nedlands Western Australia Australia; ^7^ School of Psychology The University of Western Australia Nedlands Western Australia Australia; ^8^ Office of the Vice‐Chancellor Edith Cowan University Mount Lawley Western Australia Australia; ^9^ School of Population and Global Health The University of Western Australia Nedlands Western Australia Australia

**Keywords:** gender identity, indigenous health, sexuality, social determinants of health

## Abstract

**Objective:**

To explore Aboriginal and Torres Strait Islander Lesbian Gay Bisexual Transgender Queer/questioning, and Asexual (LGBTQA+) young people's social and emotional wellbeing (SEWB) and examine associations between SEWB and positive media representation, family and community LGBTQA+ identity acceptance and experiences of racism and queerphobia.

**Study Type:**

Cross‐sectional survey.

**Setting:**

Online survey conducted between February and July 2022.

**Participants:**

Convenience sample of 619 Aboriginal and Torres Strait Islander LGBTQA+ young people (14–25 years).

**Main Outcome Measures:**

The SEWB (Connection to Mind and Emotions; Spirit, Spirituality and Ancestors; Country; Culture; Community; Family and Kinship; and Body) assessed across 15 wellbeing outcomes. The Direction of relationships between demographics, risk and protective factors and wellbeing outcomes.

**Results:**

Women had better wellbeing outcomes compared with men. Trans participants reported greater pride in their Aboriginal and Torres Strait Islander identity and more frequent Aboriginal and Torres Strait Islander LGBTQA+ event participation compared with cisgender participants. Acceptance of LGBTQA+ identity was consistently associated with positive wellbeing outcomes. Queerphobia was associated with lower Connection to Community whereas LGBTQA+ racism and relationship racism were associated with greater Connection to Culture, Country, Community, Spirit, Spirituality and Ancestors, and greater pride in Aboriginal and Torres Strait Islander identity. Participants with a disability or chronic health condition had lower Aboriginal and Torres Strait Islander community event participation, lower Connection to Country and worse physical health.

**Conclusion:**

Wellbeing can be enhanced by facilitating connection to supportive communities, fair media representation and building acceptance of LGBTQA+ identities within family and community.

## Plain Language Summary

**The known**: Aboriginal and Torres Strait Islander LGBTQA+ young people experience risk of poor health. The social and emotional wellbeing (SEWB) model provides a culturally grounded framework for assessing wellbeing.

**The new**: Acceptance of LGBTQA+ identity, fair media representation and experiences of racism were associated with higher wellbeing whereas queerphobia was associated with lower wellbeing.

**The implications**: Healthcare for Aboriginal and Torres Strait Islander LGBTQA+ young people requires consideration of multiple domains of SEWB and their experiences within multiple communities.

## 1 Introduction

Meeting Aboriginal and Torres Strait Islander Lesbian Gay Bisexual Transgender, Queer/questioning, and Asexual (LGBTQA+) young people's aspirations for healthcare requires understanding Aboriginal‐defined concepts of wellbeing. This needs to be considered alongside the unique environmental factors that arise from the intersection of being Aboriginal and/or Torres Strait Islander, queer and young. The social and emotional wellbeing (SEWB) model [1] provides an expansive view of health that prioritises connections within physical, emotional, interpersonal, cultural and spiritual domains. Grounded in Aboriginal ontology, SEWB more comprehensively describes wellbeing for Aboriginal and Torres Strait Islander young people compared with biomedical models, which focus only on physical and psychological symptoms [2, 3]. We use the SEWB model to examine the wellbeing of Aboriginal and Torres Strait Islander LGBTQA+ young people (14–25 years) who have unique risks and opportunities for achieving wellbeing [4, 5, 6]. This article is part of the 2026 *Medical Journal of Australia* Future Healthy Countdown 2030 collection [7], which examines how policy and practice can promote good physical and mental health to improve the health and wellbeing of children, young people and future generations.

The SEWB model situates wellbeing firmly within Indigenous relationality. Individuals are said to be ‘well’ when they are connected to (i.e., in relation with) Country, culture, community, family and kinship, spirit, spirituality and ancestors, body, and mind and emotions. Connection in each domain is achieved in a variety of ways [1, 8, 9, 10, 11, 12, 13, 14]. Connections to each of these domains are either interrupted or strengthened by individuals' social, political and historical context (e.g., poverty, racist government policy) [1, 2].

Growing research with Aboriginal and Torres Strait Islander LGBTQA+ people demonstrates high prevalence of psychological distress, mental health diagnoses and suicidal ideation and attempts [5] but, to our knowledge, no study to date has quantitatively explored Aboriginal and Torres Strait Islander LGBTQA+ young people's wellbeing across Aboriginal‐defined domains to gain a more comprehensive picture of their health. Doing so requires particular attention to the multiple communities and kinship systems within which Aboriginal and Torres Strait Islander LGBTQA+ youth are embedded, the positive and negative experiences within these systems, and factors supporting healthy identity development [15, 16]. Illustratively, connection to community and family and kinship networks is a key indicator of wellbeing. Transphobia and homophobia (or queerphobia) within Aboriginal and Torres Strait Islander communities is negatively associated with wellbeing [5]. In response, many Aboriginal and Torres Strait Islander LGBTQA+ people seek supportive kinship and community relationships with queer communities [16, 17]. In these contexts, connection to family or community networks that express queerphobia would signal compromised wellbeing, whereas connection with affirming communities may foster wellbeing.

This study had two aims: (1) to explore Aboriginal and Torres Strait Islander LGBTQA+ young people's wellbeing across SEWB domains, while considering the multiple communities and kinship systems in which this group of young people may be embedded, and (2) examine how connections within SEWB are related to the unique risk and protective factors for Aboriginal and Torres Strait Islander LGBTQA+ young people.

## 2 Methods

The study authorship team represents a mix of Aboriginal LGBTQA+ young people (KD, JHLH, LH), Aboriginal LGBTQA+ scholars (SLH, BHi), Aboriginal scholars (BU) and non‐Aboriginal/LGBTQA+ scholars (BHa, AL). The diversity of our combined lived experience provided us with multiple viewpoints on the study design, conduct and interpretation of the results including, crucially, the lived experience of Aboriginal LGBTQA+ young people. The study reported against the CONSolIDated critERia for strengthening the reporting of health research involving Indigenous Peoples (CONSIDER) statement [18] (Table S1) and Strengthening the Reporting of Observational Studies in Epidemiology (STROBE checklist) [19] (Table S2). Study measures, methods and analyses were pre‐registered via Open Science Framework (see Section S1, Table S3 for pre‐registered analysis plan and deviations).

### 2.1 Setting and Participants

Between February and July 2022, we conducted an online survey open to Aboriginal and Torres Strait Islander LGBTQA+ young people aged 14–25 years living in Australia. Recruitment was via advertisement on social media, partnering with LGBTQA+ health services, university Aboriginal student centres and flyers sent to all Aboriginal Community Controlled Health Organisations in Australia (148 organisations). For accessibility, participants could request to complete the survey on paper or phone.

### 2.2 Centring Aboriginal and Torres Strait Islander LGBTQA+ Young Peoples' Voices

The survey instrument emerged from a 7‐year collaboration between Aboriginal and Torres Strait Islander LGBTQA+ young people, community members and researchers (noting that many collaborators occupy multiple roles). The survey development was an iterative, Aboriginal and Torres Strait Islander LGBTQA+ led process that included:

- A previous qualitative study of how Aboriginal LGBTQA+ youth in Boorloo conceptualise their wellbeing [6]. The centrality of connection for wellbeing emerged from this review and drove the adoption of the SEWB framework for the survey.
- Identification of existing measures of the seven SEWB domains with adaptation if needed, or the development of new items.
- Extensive review and design of the survey instrument that included, but was not limited to, input from the project's Governance Committee and the Youth Advisory Group, comprising exclusively of members who are Aboriginal and LGBTQA+. Further feedback was sought from Torres Strait Islander LGBTQA+ community members.
- Development of the study pre‐registration (Section S1) and subsequent deviations (Table S3) with the Youth Advisory Group and Governance Committee alongside research team members.

This co‐design process directed the study's focus towards associations between SEWB and the independent variables listed below. Both exposure and outcome variables were selected based on the co‐design process.

### 2.3 Exposure Variables

#### 2.3.1 Fair Media Representation

Perceived fairness of media representation was assessed via agreement with the statement ‘Aboriginal and Torres Strait Islander LGBTQA+ people are often represented fairly in the media’, measured using a 5‐point Likert scale ranging from ‘Strongly Disagree’ (1) to ‘Strongly Agree’ (5). This item was developed specifically for the study.

#### 2.3.2 Acceptance of LGBTQA+ Identity

Assessed across five items developed for the study. Items asked agreement with the statement ‘I feel that me being LGBTQA+ is accepted by the following people’, where applicable, about parents/caregivers, siblings, extended family (e.g., aunties, cousins), community members and Elders. Items were measured using 5‐point Likert‐scale questions ranging from ‘Strongly Disagree’ (1) to ‘Strongly Agree’ (5). Participant's average score across the five items was used as the acceptance of LGBTQA+ identity variable.

#### 2.3.3 Microaggressions

Measure consisting of three subscales (scored separately) adapted from the LGBT People of Colour Microaggressions Scale [20]. The adapted measure was previously used with a sample of Aboriginal and Torres Strait Islander LGBTQA+ adults [21]. Following the scoring method outlined by Balsam and colleagues [20], a composite variable for each subscale was created, based on experience and impact.

##### 2.3.3.1 LGBTQA+ Racism

Four examples of LGBTQA+ racism were provided (e.g., ‘Having to educate non‐Indigenous LGBTQA+ people about race/cultural issues’), with participants asked to state whether they had experienced each of them in the last 12 months (yes/no), and if so, how much it had bothered them on a scale from ‘Not at all’ (1) to ‘Extremely’ (4).

##### 2.3.3.2 Aboriginal and Torres Strait Islander Queerphobia

Measured in the same way as LGBTQA+ racism, using four examples of Aboriginal and Torres Strait Islander queerphobia. For example, ‘Feeling like your LGBTQA+ identity is not seen in the Aboriginal and Torres Strait Islander community’ in the last 12 months (yes/no) and if so, how much it had bothered them on a scale from ‘Not at all’ (1) to ‘Extremely’ (4).

##### 2.3.3.3 Relationship Racism

Also measured in the same way as LGBTQA+ racism, but with only two examples of relationship racism. For example, ‘Feeling like non‐Indigenous LGBTQA+ people are only interested in you because of the way you look’ in the last 12 months (yes/no), and if so, how much it had bothered them on a scale from ‘Not at all’ (1) to ‘Extremely’ (4).

### 2.4 Outcome Variables

A comprehensive validated measure of SEWB for Aboriginal and Torres Strait Islander youth does not exist. As such, each of the seven SEWB domains were measured using items either created specifically for the study or adapted from pre‐existing measures of specific SEWB domains (Table S4). Domains were measured with between one and six items. The Connection to Mind and Emotions, and Connection to Community domains included items specific to the experience of Aboriginal and Torres Strait Islander LGBTQA+ young people, which do not appear in standard SEWB measures.

#### 2.4.1 Connection to Mind and Emotions

This included four items adapted from the validated Inner Peace subscale of the Growth and Empowerment Measure [22], with responses ranging from 0 [not at all true for me] to 100 [completely true for me] as measured by visual analogue. Items were averaged for a final Inner Peace score and the scale had high internal reliability (*α* = 0.869). The Connection to Mind and Emotions domain was also assessed via two additional items, both measured using 5‐point Likert scales (Strongly disagree = 1 to Strongly agree = 5), with one measuring pride in LGBTQA+ identity and the other measuring pride in Aboriginal and Torres Strait Islander identity. These items were developed specifically for the study.

#### 2.4.2 Connection to Family and Kinship

A single validated item from the Aboriginal Cultural Engagement Survey [23], asking: ‘I have a strong sense of kinship/family links’ (1 = Strongly disagree; 5 = Strongly agree).

#### 2.4.3 Connection to Spirit, Spirituality and Ancestors

One validated item [23]: ‘I have a strong sense of connection to my spirituality and ancestors’ (0 = Not at all true for me; 100 = Completely true for me).

#### 2.4.4 Connection to Country

One validated item from the Aboriginal Cultural Engagement Survey [23] assessed Connection to Country: ‘I feel like I belong to my people's land/waters’, with responses ranging from ‘Not at all’ (1) to ‘A lot’ (4).

#### 2.4.5 Connection to Culture

Two items adapted from the Aboriginal Cultural Engagement Survey [23] asking how often they spent time learning about Culture and whether they participated in cultural practices (‘Never’ [0] to ‘Very often’ [4]), were asked. Scores on the two items were averaged.

#### 2.4.6 Connection to Community

Two validated questions adapted from the Aboriginal Cultural Engagement Survey [23] asked whether participants felt they belonged to a given community (Aboriginal and/or Torres Strait Islander; LGBTQA+ community; Aboriginal and/or Torres Strait Islander and LGBTQA+ community; ‘Strongly disagree’ [1] to ‘Strongly agree’ [5]) and whether they participated in events in each of these communities (‘Never’ [0] to ‘Very often’ [4]).

#### 2.4.7 Connection to Body

One validated item measuring self‐rated physical health [24] (0 = Extremely bad; 50 = Average; 100 = Extremely good) was asked. One item assessing whether alcohol and drug use had impacted participants' lives (1 = ‘No, not at all’; 2 = ‘No, not really’; 3 = ‘Neither yes nor no’; 4 = ‘Yes, a bit’; 5 = ‘Yes, a lot’) was asked of participants who had indicated earlier in the survey that they used alcohol or drugs. Each item was used as separate indicator of Connection to Body.

### 2.5 Demographic Variables

Demographic covariates were selected based on prior literature and discussions with the study Youth Advisory Group. Age was derived from date of birth and date of survey completion. Disability status was assessed via asking ‘Do you have a long‐term health problem or disability’? (0 = no; 1 = yes). Free‐text questions were used for gender and sexuality with responses coded initially by SLH with codes then reviewed by the Youth Advisory Group and remaining authors. Disagreements on codes were resolved by reaching consensus through discussion, where Aboriginal LGBTQA+ youth perspective was prioritised (Table S5). Gender was coded into man, woman and non‐binary/another gender/questioning. Sexuality was coded into gay/lesbian, bisexual/pansexual/omnisexual and asexual/queer/questioning/another sexuality. Gender diversity was derived from the question ‘Is your gender different from the gender presumed on your birth? i.e., the one on your original birth certificate’. Responses were coded into transgender, unsure or cisgender.

### 2.6 Data Analysis

Data cleaning procedures are outlined in the study pre‐registration section of the Supporting Information (Section S1). A total of 316 invalid responses were removed from the analysis. Among the remaining participants, data was missing in a monotone pattern, reflecting participant drop‐off.

As per our pre‐registration plan, we tested whether variables across the SEWB domains could be used to create composite SEWB scores. Participants' scores for each item were standardised using *z*‐scores and then averaged to create an overall SEWB scale. This scale showed adequate internal reliability (*α* = 0.768). However, we opted to keep each SEWB domain separate in our analyses given that the original model proposes that each domain is separate but related [1]. Likewise, although there are only seven SEWB domains, some domains required having more than one indicator (Connection to Mind and Emotions, Connection to Community, Connection to Body) to ensure they were adequately measured and inclusive of youth and LGBTQA+ experiences (i.e., adding Aboriginal and LGBTQA+ community items in the Connection to Community domain and adding the pride in identity items to the Connection to Mind and Emotions domain). This resulted in 15 wellbeing outcomes across the seven SEWB domains. Pearson correlations between all variables were used to assess potential multicollinearity (Tables S6–S8).

A series of multivariable linear regression models were fit for each of the 15 wellbeing outcomes to assess the strength and direction of associations between exposure and demographic variables. Outcomes scored on a scale of 0–100 (Inner Peace, self‐reported physical health and Connection to Spirit, Spiritualty and Ancestors) were rescaled by dividing by 10 to assist with model convergence. As this is an exploratory analysis intended to describe relationships between exposures and outcomes, *p* values are not reported. Our determination of meaningful associations between the exposure variables and each of the wellbeing outcomes was based on the unstandardised model coefficients and uncertainty represented in their 95% confidence intervals (CIs), and whether the magnitude of the coefficients represented a meaningful change in the context of each SEWB item's original scale.

All models were run in Mplus V9 [25] using robust standard errors to account for outcome non‐normality. To mitigate the impact of missing data, Full Information Maximum Likelihood method (FIML) was used as it produces estimates using all observed data in the sample [26].

### 2.7 Ethics Statement

Ethical approval was received from the Western Australian Aboriginal Health Ethics Committee (WAAHEC), the Human Research Ethics Committee of Northern Territory Health and Menzies School of Health Research (NT HREC), the Central Australian Health Research Ethics Committee (CAHREC), the Aboriginal Health Research Ethics Committee (AHREC), the Australian Institution for Aboriginal and Torres Strait Islander Studies (AIATSIS), the Aboriginal Health and Medical Research Council (AH&MRC), the University of Western Australia and ACON. All participants provided informed consent prior to data collection.

## 3 Results

Table 1 provides percentages/mean scores and standard deviations for all demographic, exposure and outcome variables.

**TABLE 1:** Descriptive statistics for participant demographics and study variables.

	Mean (standard deviation) or proportion (%)	Range	*N*
Age	17.51 (2.86)	14–25	617
Missing			2
Gender			614
Non‐binary/another gender	40.7%		250
Woman	42.8%		263
Man	16.4%		101
Missing	—		5
Gender diversity			612
Trans	46.2%		283
Unsure	8.5%		52
Cisgender	45.3%		277
Missing	—		7
Sexuality^a^			617
Bisexual/pansexual/omnisexual	56.6%		349
Asexual/queer/questioning/other	22.4%		138
Gay/lesbian	21.1%		130
Missing	—		2
Disability			612
No disability or chronic health condition	60.3%		369
Disability or chronic health condition	39.7%		243
Missing	—		7
Exposures			
Fair media representation	1.78 (0.82)	1–5	610
Aboriginal and Torres Strait Islander queerphobia	2.52 (1.41)	0–5	415
LGBTQA+ racism	2.70 (1.48)	0–5	422
Relationship racism	0.90 (1.48)	0–5	412
Acceptance of LGBTQA+ identity	3.38 (0.76)	1–5	612
Outcomes			
Connection to mind and emotion			
Inner peace	4.17 (2.43)	0–10	464
Pride LGBTQA+	4.44 (0.95)	1–5	412
Pride Aboriginal and Torres Strait Islander	4.48 (0.97)	1–5	411
Connection to family and kinship			
Family and kinship	3.23 (1.28)	1–5	604
Connection to culture			
Culture	2.00 (1.20)	0–4	484
Spirit, spirituality and ancestors			
Spirit	4.22 (3.22)	0–10	464
Connection to country			
Land and waters	2.40 (0.94)	1–4	590
Connection to community			
Belonging LGBTQA+ community	4.16 (1.05)	1–5	394
Belonging Aboriginal & Torres Strait Islander community	3.34 (1.28)	1–5	540
Belonging Aboriginal & Torres Strait Islander and LGBTQA+ community	2.84 (1.41)	1–5	534
Participation in LGBTQA+ events	1.70 (1.32)	0–4	547
Participation in Aboriginal and Torres Strait Islander events	2.32 (1.34)	0–4	546
Participation in Aboriginal & Torres Strait Islander and LGBTQA+ events	0.88 (1.19)	0–4	531
Connection to body			
Physical health	5.05 (2.05)	0–10	465
Substance use impact	2.42 (1.23)	1–5	236

*Note:* The following acronyms are used: LGBTQA+ = lesbian, gay, bisexual, trans, queer/questioning, asexual. Valid percentages are reported.

^a^ Before non‐binary and ‘another gender’ were combined: Non‐binary *n* = 217 and ‘another gender’/questioning *n* = 33. Before asexual/queer/questioning/other were combined: Asexual *n* = 45, queer = 48, questioning/other sexuality = 45.

### 3.1 Participant Characteristics

The total number of participants was 619 Aboriginal and Torres Strait Islander LGBTQA+ young people with a mean age of 17.5 years. Of these, 42.8% were women, 40.7% were non‐binary and 46.2% had a gender different from that presumed at birth. Regarding sexuality, 56.6% identified as bisexual, pansexual or omnisexual.

### 3.2 Exposures

Most participants did not feel that Aboriginal and Torres Strait Islander LGBTQA+ people were often fairly represented in the media. On average, participants somewhat agreed that their LGBTQA+ identity was accepted by family, community and Elders; however, most participants had been impacted by more than one instance of LGBTQA+ racism and Aboriginal and Torres Strait Islander queerphobia in the past 12 months. Relationship racism was not commonly experienced by participants.

### 3.3 SEWB

Within the Connection to Mind and Emotions domain, most participants felt pride in their LGBTQA+ and Aboriginal and Torres Strait Islander identities; however, they experienced a moderate level of inner peace. Most participants agreed that they experienced Connection to Family and Kinship and sometimes learned about or participated in culture (Connection to Culture). On average, participants strongly agreed that they experienced Connection to Spirit, Spirituality and Ancestors and Connection to Country. Within the domain of Connection to Community, on average participants rarely or never attended LGBTQA+ or Aboriginal and Torres Strait Islander LGBTQA+ community events. In contrast, participants often attended Aboriginal and Torres Strait Islander events. Most participants felt a strong sense of belonging to an LGBTQA+ community, an Aboriginal and Torres Strait Islander community and an Aboriginal and Torres Strait Islander LGBTQA+ community.

### 3.4 Multivariable Regression Models' Goodness of Fit

Unstandardised beta values, 95% confidence intervals and *R* ^2^ values are presented in Tables 2, 3, 4. The model estimates represent the correlation between a given covariate and SEWB outcome when all other covariates are held constant. As such, estimates for each exposure variable should not be taken as evidence of single effects. Models explained between 8% and 22% of variance in the wellbeing outcomes. Figure 1 provides a visual representation of model estimates and is intended to show the patterns of point estimates and CIs across models rather than give precise detail on estimates. Figure 1 demonstrates that there was greatest precision (narrowest 95% CI ranges) in estimates for age, disability, fair media representation, acceptance of LGBTQA+ identity, Aboriginal and Torres Strait Islander queerphobia, LGBTQA+ racism and relationship racism across the SEWB outcomes.

**TABLE 2:** Unstandardised coefficients, 95% confidence intervals, and *R* ^2^ values for inner peace, pride in LGBTQA+ identity, pride in Aboriginal and Torres Strait Islander identity, connection to spirit, spirituality, ancestors and kinship models.

	Connection to mind and emotions			Connection spirit, spirituality and ancestors	Connection to family and kinship
	Model 1 (inner peace)	Model 2 (LGBTQA+ pride)	Model 3 (Aboriginal and Torres Strait Islander pride)	Model 4 (spirit and ancestors)	Model 5 (kinship)
	*B* (95% CIs)	*B* (95% CIs)	*B* (95% CIs)	*B* (95% CIs)	*B* (95% CIs)
*R* ^2^	0.228	0.101	0.141	0.189	0.158
Gender (ref = man)					
Woman	0.325 (−0.342, 0.993)	−0.003 (−0.350, 0.344)	0.458 (0.139, 0.777)	1.336 (0.115, 2.264)	0.380 (0.047, 0.714)
Non‐binary or another gender	−0.225 (−0.880, 0.430)	0.217 (−0.068, 0.502)	−0.031 (−0.324, 0.261)	0.720 (−0.147, 1.587)	−0.038 (−0.333, 0.257)
Gender diversity (ref = cis)					
Trans	−0.152 (−0.773, 0.468)	0.163 (−0.161, 0.486)	**0.272 (0.003, 0.541)**	0.263 (−0.596, 1.123)	−0.045 (−0.343, 0.253)
Unsure or prefer not to say	0.182 (−0.710, 1.074)	0.003 (−0.423, 0.428)	0.037 (−0.39, 0.464)	−0.734 (−1.869, 0.401)	0.114 (−0.291, 0.519)
Sexuality (ref = gay/lesbian)					
Bisexual/pansexual/omnisexual	−0.179 (−0.692, 0.334)	−0.027 (−0.253, 0.200)	−0.079 (−0.298, 0.140)	0.259 (−0.447, 0.965)	−0.034 (−0.273, 0.205)
Asexual, queer or another sexuality	−0.201 (−0.791, 0.39)	−0.053 (−0.312, 0.206)	−0.221 (−0.493, 0.140)	−0.052 (−0.846, 0.742)	−0.208 (−0.502, 0.085)
Age	**0.240 (0.167, 0.313)**	0.014 (−0.015, 0.043)	0.031 (−0.002, 0.064)	**0.227 (0.125, 0.328)**	0.010 (−0.027, 0.046)
Disability	**−0.781 (−1.206, −0.357)**	0.147 (−0.031, 0.325)	−0.154 (−0.361, 0.053)	**−0.770 (−1.354, −0.186)**	−0.166 (−0.376, 0.043)
Media frequency	−0.196 (−0.419, 0.028)	0.008 (−0.116, 0.132)	−0.069 (−0.195, 0.058)	−0.065 (−0.424, 0.293)	−0.044 (−0.168, 0.080)
Acceptance average	**0.871 (0.592, 1.150)**	**0.286 (0.137, 0.435)**	**0.168 (0.038, 0.297)**	**0.765 (0.387, 1.144)**	**0.526 (0.396, 0.656)**
Aboriginal queerphobia	−0.076 (−0.245, 0.092)	0.009 (−0.071, 0.089)	0.005 (−0.066, 0.076)	**−0.281 (−0.511, −0.051)**	−0.072 (−0.164, 0.020)
LGBTQA+ racism	0.070 (−0.097, 0.238)	−0.028 (−0.086, 0.030)	**0.167 (0.095, 0.240)**	**0.500 (0.277, 0.723)**	0.056 (−0.038, 0.150)
Relationship racism	−0.123 (−0.274, 0.029)	−0.023 (−0.099, 0.052)	−0.051 (−0.113, 0.012)	**0.235 (0.017, 0.452)**	−0.007 (−0.094, 0.081)

*Note:* Model results did not change substantially when ‘asexual’ and ‘queer/another sexuality/questioning’ or ‘non‐binary’ and ‘another gender/questioning’ were included as separate variables. Bolded estimates indicate where 95% CIs do not cross zero.

**TABLE 3:** Unstandardised coefficients, 95% confidence intervals, and *R* ^2^ values for connection to culture, community events and community belonging models.

	Connection to culture	Connection to community			
	Model 6 (culture)	Model 7 (LGBTQA+ events)	Model 8 (Aboriginal and Torres Strait Islander events)	Model 9 (aboriginal and LGBTQA+ events)	Model 10 (LGBTQA+ belonging)
	*B* (95% CIs)	*B* (95% CIs)	*B* (95% CIs)	*B* (95% CIs)	*B* (95% CIs)
*R* ^2^	0.208	0.112	0.203	0.108	0.104
Gender (ref = man)					
Woman	0.418 (0.059, 0.777)	−0.020 (−0.383, 0.343)	**0.679 (0.303, 1.054)**	**0.542 (0.213, 0.871)**	−0.082 (−0.420, 0.256)
Non‐binary or another gender	0.064 (−0.237, 0.364)	**0.359 (0.014, 0.703)**	**0.462 (0.123, 0.802)**	0.090 (−0.196, 0.376)	0.173 (−0.102, 0.449)
Gender diversity (ref = cis)					
Trans	0.040 (−0.279, 0.360)	0.267 (−0.083, 0.617)	−0.131 (−0.469, 0.208)	**0.572 (0.238, 0.905)**	0.271 (−0.026, 0.569)
Unsure or prefer not to say	0.042 (−0.318, 0.402)	−0.221 (−0.673, 0.231)	−0.290 (−0.684, 0.103)	**0.386 (0.012, 0.760)**	0.023 (−0.352, 0.398)
Sexuality (ref = gay/lesbian)					
Bisexual/pansexual/omnisexual	0.139 (−0.084, 0.362)	0.135 (−0.150, 0.420)	0.143 (−0.122, 0.408)	0.210 (−0.039, 0.458)	0.009 (−0.219, 0.236)
Asexual, queer or another sexuality	−0.156 (−0.408, 0.096)	−0.013 (−0.332, 0.305)	−0.072 (−0.382, 0.237)	−0.139 (−0.408, 0.131)	−0.177 (−0.449, 0.096)
Age	0.007 (−0.027, 0.041)	0.004 (−0.036, 0.044)	0.026 (−0.011, 0.063)	0.023 (−0.015, 0.061)	−0.059 (−0.094, −0.024)
Disability	−0.174 (−0.373, 0.024)	0.144 (−0.084, 0.372)	**−0.434 (−0.662, −0.206)**	0.081 (−0.127, 0.288)	0.069 (−0.116, 0.255)
Media frequency	0.011 (−0.103, 0.124)	−0.042 (−0.170, 0.085)	0.029 (−0.098, 0.156)	**0.131 (0.010, 0.251)**	−0.011 (−0.130, 0.108)
Acceptance average	**0.166 (0.039, 0.293)**	**0.336 (0.186, 0.486)**	**0.252 (−0.097, 0.407)**	**0.240 (0.098, 0.383)**	**0.223 (0.094, 0.352)**
Aboriginal queerphobia	−0.071 (−0.148, 0.006)	0.090 (−0.010, 0.190)	**−0.157 (−0.249, −0.066)**	**−0.093 (−0.184, −0.002)**	0.041 (−0.037, 0.119)
LGBTQA+ racism	**0.249 (0.176, 0.322)**	−0.041 (−0.140, 0.059)	**0.272 (0.179, 0.365)**	0.083 (−0.010, 0.175)	−0.057 (−0.126, 0.012)
Relationship racism	**0.123 (0.046, 0.200)**	**0.112 (0.017, 0.207)**	**0.093 (0.004, 0.183)**	**0.102 (0.006, 0.199)**	0.061 (−0.011, 0.133)

*Note:* Model results did not change substantially when ‘asexual’ and ‘queer/another sexuality/questioning’ or ‘non‐binary’ and ‘another gender/questioning’ were included as separate variables. Bolded estimates indicate where 95% CIs do not cross zero.

**TABLE 4:** Unstandardised coefficients, 95% confidence intervals, and *R* ^2^ values for community belonging, connection to country, physical health and substance use impact models.

	Connection to community		Connection to country	Connection to body	
	Model 11 (Aboriginal and Torres Strait Islander belonging)	Model 12 (aboriginal and LGBTQA+ belonging)	Model 13 (land and waters)	Model 14 (physical health)	Model 15 (substance use impact)
	*B* (95% CIs)	*B* (95% CIs)	*B* (95% CIs)	*B* (95% CIs)	*B* (95% CIs)
*R* ^2^	0.138	0.118	0.114	0.083	0.087
Gender (ref = man)					
Woman	0.270 (−0.111, 0.651)	−0.160 (−0.611, 0.291)	0.142 (−1.080, 0.393)	−0.005 (−0.077, 0.066)	−0.052 (−0.607, 0.503)
Non‐binary or another gender	−0.016 (−0.348, 0.315)	−0.109 (−0.507, 0.288)	0.047 (−0.198, 0.292)	−0.187 (−0.813, 0.438)	−0.166 (−0.674, 0.342)
Gender diversity (ref = cis)					
Trans	0.320 (−0.021, 0.662)	0.250 (−0.176, 0.676)	0.067 (−0.171, 0.306)	−0.067 (−0.699, 0.564)	−0.016 (−0.497, 0.466)
Unsure or prefer not to say	−0.015 (−0.412, 0.382)	0.050 (−0.395, 0.495)	−0.172 (−0.484, 0.139)	0.053 (−0.792, 0.898)	−0.354 (−0.890, 0.182)
Sexuality (ref = gay/lesbian)					
Bisexual/pansexual/omnisexual	0.062 (−0.205, 0.328)	0.283 (−0.024, 0.591)	0.166 (−0.025, 0.357)	−0.097 (−0.596, 0.402)	−0.045 (−0.473, 0.384)
Asexual, queer or another sexuality	−0.144 (−0.456, 0.168)	0.041 (−0.315, 0.398)	0.005 (−0.220, 0.230)	0.059 (−0.515, 0.634)	0.087 (−0.406, 0.581)
Age	0.022 (−0.016, 0.060)	−0.038 (−0.080, 0.003)	0.018 (−0.012, 0.048)	−0.005 (−0.077, 0.066)	0.057 (0.004, 0.109)
Disability (ref = disability or health condition)	−0.351 (−0.580, −0.122)	−0.108 (−0.360, 0.143)	**−0.177 (−0.340, −0.014)**	**−0.891 (−1.318, −0.463)**	0.140 (−0.192, 0.471)
Media frequency	0.088 (−0.050, 0.226)	**0.209 (0.042, 0.377)**	−0.006 (−0.101, 0.088)	−0.081 (−0.329, 0.166)	0.046 (−0.192, 0.273)
Acceptance average	**0.241 (0.081, 0.402)**	**0.313 (0.148, 0.478)**	**0.139 (0.037, 0.241)**	**0.409 (0.110, 0.708)**	−0.171 (−0.392, 0.051)
Aboriginal queerphobia	−0.206 (−0.296, −0.116)	−0.169 (−0.272, −0.065)	−0.048 (−0.115, 0.020)	−0.012 (−0.182, 0.158)	0.016 (−0.10, 9.141)
LGBTQA+ racism	**0.193 (0.103, 0.283)**	0.100 (−0.001, 0.200)	**0.179 (0.114, 0.244)**	0.127 (−0.041, 0.296)	**0.135 (0.00, 1.269)**
Relationship racism	**0.114 (0.031, 0.198)**	**0.185 (0.085, 0.285)**	0.038 (−0.025, 0.102)	−0.076 (−0.240, 0.089)	−0.002 (−0.115, 0.111)

*Note:* Model results did not change substantially when ‘asexual’ and ‘queer/another sexuality/questioning’ or ‘non‐binary’ and ‘another gender/questioning’ were included as separate variables. Bolded estimates indicate where 95% CIs do not cross zero.

**FIGURE 1:**
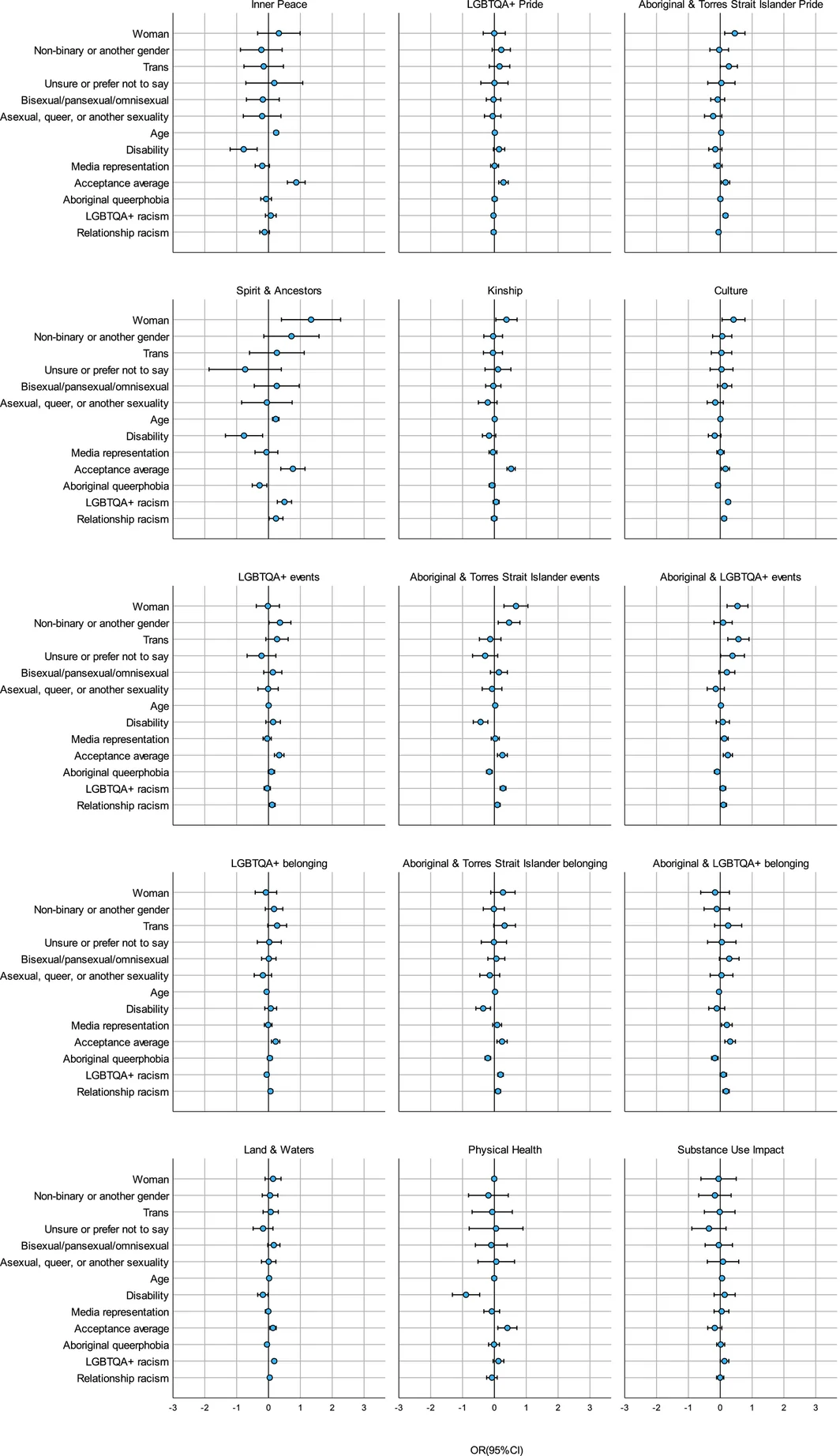
Unstandardised coefficients and 95% upper and lower confidence intervals for SEWB models. CI = confidence interval, LGBTQA+ = lesbian, gay, bisexual, trans, queer/questioning, asexual.

### 3.5 Associations Between Demographic Factors and SEWB Outcomes

Compared with men, women had higher Connection to Family and Kinship, Connection with Spirit, Spiritualty and Ancestors, Connection to Culture, greater pride in their Aboriginal and Torres Strait Islander identity and greater participation in some community events.

Participants who were non‐binary/another option/questioning had more frequent participation in LGBTQA+ community events and Aboriginal and Torres Strait Islander community events, compared with participants who were men. Trans participants had greater pride in their Aboriginal and Torres Strait Islander identity and more frequent participation in Aboriginal and Torres Strait Islander LGBTQA+ community events compared with cisgender participants. Participants who were unsure of their gender diversity/preferred not disclose had more frequent Aboriginal and Torres Strait Islander LGBTQA+ event participation compared with cisgender participants.

Participants with a disability or chronic health condition had lower scores in five of the 15 outcomes, across five SEWB domains, compared with participants without a disability or chronic health condition. Older participants had higher inner peace and connection to spirit, spirituality and ancestors.

### 3.6 Associations Between Exposure Variables and SEWB Outcomes

#### 3.6.1 Fair Media Representation

The perception of fair media representation was only associated with one SEWB domain (Connection to Community). Participants who perceived greater frequency of fair media representation had greater participation in Aboriginal and Torres Strait Islander LGBTQA+ community events and had higher feelings of belonging to an Aboriginal and Torres Strait Islander LGBTQA+ community.

#### 3.6.2 Acceptance of LGBTQA+ Identity

Acceptance of LGBTQA+ identity was the most consistent factor associated with SEWB outcomes, being positively associated with 14 of the 15 outcomes (except for substance use impact) with small to moderate effect sizes.

#### 3.6.3 LGBTQA+ Racism

Higher levels of LGBTQA+ racism were associated with higher wellbeing scores across 7 of the 15 SEWB outcomes relating to Aboriginal and Torres Strait Islander community connection and pride, Connection to Spirit, Spirituality, and Ancestors, Connection to Culture, Connection to Land and Waters, and reduced substance use impact.

#### 3.6.4 Aboriginal and Torres Strait Islander Queerphobia

Greater Aboriginal and Torres Strait Islander queerphobia was associated with lower SEWB across two of the domains. Participants who reported higher queerphobia had lower levels of Connection to Spirit, Spiritualty and Ancestors, and lower connection to an Aboriginal and Torres Strait Islander and Aboriginal and Torres Strait Islander LGBTQA+ community.

#### 3.6.5 Relationship Racism

There were positive associations between levels of relationship racism and SEWB outcomes. Specifically, higher levels of relationship racism were associated with higher levels of Connection to Spirit, Spirituality and Ancestors, Connection to Culture, Connection to Community (all items except feelings of belonging to an LGBTQA+ community).

## 4 Discussion

Leveraging an Aboriginal‐defined model of SEWB with an additional lens of LGBTQA+ and youth perspectives, this study mapped Aboriginal and Torres Strait Islander LGBTQA+ youth wellbeing to include connections with families, communities and non‐physical entities (e.g., Connection to Culture, Connection to Spirit, Spirituality and Ancestors). In general, study participants had moderate to high levels of SEWB, with SEWB outcomes tending to be higher when acceptance of LGBTQA+ identities were reported, and lower when experiences of queerphobia within Aboriginal and Torres Strait Islander communities were reported.

Our findings support longstanding community concerns for risks associated with SEWB among Aboriginal and Torres Strait Islander LGBTQA+ young people [4, 27, 28, 29]. The current findings are also consistent with previous research that points to associations between LGBTQA+ identity acceptance and wellbeing. Across Australia, programs for Aboriginal and Torres Strait Islander LGBTQA+ people have focused on building connections within Aboriginal and Torres Strait Islander LGBTQA+ communities (i.e., Yarns around the campfire, 2Spirits, The BlaQ Retreat, BlaQ Aboriginal Corporation). This focus on community connection is not surprising given the protective role that strong community connections play in sustaining SEWB. More recently, a program that facilitates connections between Aboriginal and Torres Strait Islander LGBTQA+ people and supportive and accepting Elders has shown promising results in improving participants' perceived acceptance of LGBTQA+ identities within their community (unpublished data).

A concerning finding was that participants who experienced greater Aboriginal and Torres Strait Islander queerphobia also reported lower SEWB across several domains. This finding is consistent with previous qualitative research where Aboriginal and Torres Strait Islander LGBTQA+ people rated queerphobia within their own communities as a reason for not ‘coming out’ or for leaving their community [4, 5]. There is substantial work occurring across countries within Australia to tackle community‐level queerphobia including the ‘Rainbow Mob are Our Mob’ campaign and training developed by the Victorian Aboriginal Community Controlled Health Organisation. Our current findings add weight to the argument that continuation and expansion of these programs is an essential component of supporting Aboriginal and Torres Strait Islander LGBTQA+ young peoples' SEWB.

### 4.1 Limitations

Although this study is based on the largest sample of Aboriginal and Torres Strait Islander LGBTQA+ youth, the convenience sampling approach means that our findings may not be generalisable to the target population. We also cannot draw conclusions regarding causal or bi‐directional associations between exposures and SEWB outcomes due to the cross‐sectional nature of the study.

Relatedly, while the scope of this study was exploratory, without a causal framework that describes how the covariates and outcomes are related, the model estimates may be biased, such as from inadvertent conditioning on a collider or failure to control for a confounder. Future research should closely explore the associations between each set of SEWB outcomes and specific environmental factors via longitudinal designs. Such design would also allow researchers to unpack our somewhat counter‐intuitive finding that participants who experienced higher LGBTQA+ racism and relationship racism had better SEWB outcomes. Although previous cross‐sectional research with Aboriginal and Torres Strait Islander LGBTQA+ adults has found similar associations [21], study designs that allow researchers to test whether participants with higher SEWB are more likely to report racism or whether participants who experience racism actively seek out opportunities to bolster their SEWB are needed. Second, many of the items used to measure the SEWB domains had to be developed for the study or had not been previously validated in a sample of young people. There have been continued calls from SEWB practitioners for the development of a comprehensive SEWB measure [30]. Such a measure would help future researchers determine whether each of the seven SEWB domains should be assessed separately or whether there is support for superordinate domains. Our current findings highlight the need for any future SEWB measure development to consider the measurement of factors salient to the developmental experiences of young people (e.g., our inclusion of pride in identity as an indicator of Connection to Mind and Emotions) as well as LGBTQA+ specific experiences (e.g., measuring Connection to Community via feelings of belonging to an Aboriginal and Torres Strait Islander LGBTQA+ community).

## 5 Conclusion

Our study provides initial evidence of the factors associated with SEWB among a national sample of Aboriginal and Torres Strait Islander LGBTQA+ young people. By using the SEWB model, we present an Indigenous‐informed analysis of health for this group of young people, providing an analysis that goes beyond the binary of ‘diseased/not diseased’. Explicit in the SEWB model is the role of connection to each domain. This relational understanding of health asserts that health is not something that can be given or received. Rather, health is an ongoing and dynamic relationship with one's environment. This echoes the interconnectedness and relationality of Aboriginal and Torres Strait Islander cultures [31] and presents a decolonial alternative to existing Australian healthcare models.

## Author Contributions

Conceptualisation: B.U., S.L.‐H., K.D., J.H.L.H., B.Hi., L.H., Y.P. and A.L. Data curation: B.U., S.L.‐H. and A.L. Formal analysis: B.U. Funding acquisition: B.U., B.Hi., Y.P. and A.L. Investigation: B.U., S.L.‐H., L.H., Y.P. and A.L. Methodology: B.U., S.L.‐H., B.Ha., K.D., J.H.L.H., B.Hi., Y.P. and A.L. Project administration: B.U., S.L.‐H., L.H., Y.P. and A.L. Supervision: B.U., B.Hi., Y.P. and A.L. Visualisation: S.L.‐H. and B.Ha. Writing – original draft: B.U. Writing – reviewing and editing: S.L.‐H., B.Ha., K.D., J.H.L.H., L.H. and A.L.

## Funding

This work was supported by the National Health and Medical Research Council (NHMRC) (grant number APP1157377). This manuscript was completed while Shakara Liddelow‐Hunt was in receipt of an Aboriginal and Torres Strait Islander Research Scholarship from the University of Western Australia. Ashleigh Lin is an NHMRC Investigator Grant holder (grant number 2010063).

## Disclosure

Not commissioned; externally peer reviewed.

## Conflicts of Interest

The authors declare no conflicts of interest.

## Supporting information


**Table S1:** How this study addresses the CONSIDER checklist (Huria et al. 2019).
**Table S2:** STROBE Statement—Checklist of items that should be included in reports of cross‐sectional studies.
**Table S3:** Record of deviations from pre‐registration. Record format based on Willroth & Atherton, 2024.
**Table S4:** Full list of study exposure variable items and wellbeing outcome items.
**Table S5:** Coding framework for open‐ended gender and sexuality questions.
**Table S6:** Pearson correlations between demographics and exposure variables.
**Table S7:** Pearson correlations between exposure variables and wellbeing outcomes.
**Table S8:** Pearson correlations between demographics and exposure variables.
**Section S1:** Survey pre‐registration.

## Data Availability

The data for this study are not publicly available. Requests for analyses of the data, conducted by the research team, will be decided on a case‐by‐case basis by an Aboriginal and Torres Strait Islander LGBTQA+SB (Lesbian, Gay, Bisexual, Transgender, Intersex, Queer, Asexual, Sistergirl, Brotherboy, plus others) Research Governance. Contact the corresponding author for further information.
