# Supplementary material for: Factors Associated With Social and Emotional Wellbeing Among Aboriginal and Torres Strait Islander LGBTQA+ Young People
Source: Med J Aust. 2026 Nov 11;224(11):e70333. doi: 10.5694/mja2.70333 (PMC13754534; doi:10.5694/mja2.70333)
Supplement: Supplementary file 1 [file MJA2-224-0-s001.pdf]

MJA
The Medical Journal of Australia

## Supporting Information

## Supplementary material

**This appendix was part of the submitted manuscript and has been peer reviewed.**
**It is posted as supplied by the authors.**

Appendix to: Uink B, Liddelow-Hunt S, Harrap B, et al. Factors associated with social and emotional wellbeing among Aboriginal and Torres Strait Islander LGBTQA+ young people. *Med J Aust* 2026; doi: 10.5694/mja2.70333.

## SUPPLEMENTARY MATERIALS

### Section S1: Survey pre-registration

***Walkern Katatdjin National Survey: Aboriginal and Torres Strait Islander LGBTQA+ young peoples' social emotional wellbeing and mental health***

**Metadata**

**Contributors**

Bep Uink, Shakara Liddelow-Hunt, Ashleigh Lin, Yael Perry, Mirella Wilson, Braden Hill, Kate Daglas, James Hector Laurent Hill

**Description**

The purpose of this study is to investigate risk and protective factors for social emotional wellbeing (SEWB) and mental health among Aboriginal and Torres Strait Islander Lesbian, Gay, Bisexual, Transgender, Queer/Questioning, Asexual, and other gender or sexuality diverse (LGBTQA+) young people (14-25 years). Intersectionality theory (Crenshaw, 1991) proposes that individuals who occupy multiple marginalised identities are not captured by initiatives aimed at addressing only one form of disadvantage. Further, Minority Stress Theory (Meyer et al., 2003) proposes that, among individuals with more than one marginalised identity, those who experience discrimination and stressors associated with both identities will experience poorer wellbeing outcomes compared to those who experience stressors associated with only one marginalised identity. There is concern among Aboriginal and Torres Strait Islander communities that Aboriginal and Torres Strait Islander LGBTQA+ young people occupy three social identities- Indigenous, LGBTQA+, and young- that are made vulnerable to suicide, high psychological distress and poor SEWB (Uink et al., 2020; Dudgeon et al., 2015). Some Aboriginal and Torres Strait Islander LGBTQA+ young people report strong SEWB (Liddelow-Hunt et al., 2021; Hill et al., 2021) although studies with this population thus far have been limited to small samples and distinct geographical regions. There is no widely available evidence on the risk and protective factors associated with Aboriginal and Torres Strait Islander LGBTQA+ young peoples' SEWB and mental health across a diverse range of participants from regions across Australia. This survey study will examine novel correlates (e.g., centrality of Aboriginal and LGBTQA+ identity, community acceptance) of SEWB as well as test pre-established correlates (e.g., microaggressions, family acceptance) of psychological distress found in international studies of LGBTQA+ youth populations.

**Registration Type**

Pre-Registration in Social Psychology (van 't Veer & Giner-Sorolla, 2016): Pre-Registration

**Registry**

OSF Registries

**Date Created**

Aug 12, 2022, 5:31 PM

**Date registered**

Aug 12, 2022, 5:31 PM

**Subjects**

Social and Behavioral Sciences

**Tags**

LGBTQ+; adolescent; Indigenous; social emotional wellbeing; psychological distress; intersectionality

**Affiliated Institutions**

No affiliated institutions

**Funder Names**

No information

**Language**

No language

**Associated project**

https://osf.io/tc3x9

**internet archive link**

https://archive.org/details/osf-registrations-dnj9p-v1

**License**

No license

**Registration DOI**

10.17605/OSF.IO/DNJ9P

## A. Hypotheses - Essential elements

**Description of essential elements**

**Describe the (numbered) hypotheses in terms of directional relationships between your (manipulated or measured) variables. Updated**

Given the novelty of this field the majority of our planned analyses are exploratory in nature. We have set confirmatory hypotheses when there is evidence of a well-established relation between two variables in a similar population (e.g., ethnic minority LGBTQA+ youth) or where we are directly assessing a prediction laid out by theory. Confirmatory hypotheses are indicated where appropriate. Study 1: Research Question. What are the risk and protective factors for SEWB in Aboriginal and Torres Strait Islander LGBTQA+ young people? Our definition of SEWB is based on Gee et al's. (2014) SEWB model and refers to the connections that individuals have to seven domains: Country, Community, Culture, Family/Kinship, Spirit/Ancestors, Mind/Emotions, and Body. We assess each domain via items that were developed specifically for this survey or were adapted from previously validated measures. Because this is a new measure of SEWB we will first assess internal reliability of each sub-scale representing each domain (with the exception of domains that are measured via 1 item). We will then test associations between putative risk and protective factors with each domain as well as with a Total SEWB score (if internal reliability is appropriate). Specifically, we will explore the relations between participants' SEWB scores and following putative protective factors; i) Positive Aboriginal and Torres Strait LGBTQA+ media representation ; ii) parental/caregiver acceptance; iii) sibling acceptance; iv) acceptance from extended family; v) acceptance from Aboriginal and Torres Strait Islander community; vi) Acceptance from Elders; vii) Aboriginal and LGBTQA+ identity centrality; viii) pride in Aboriginal and Torres Strait Islander identity; ix) pride in LGBTQA+ identity. Microaggressions represent daily, sometimes subtle, forms of discrimination based on one's minority status. Previous research shows that microaggressions against LGBTQ+ peoples in ethnic minority communities, race-based microaggressions in LGBTQA+ communities, and race-based microaggressions within romantic relationships are associated with psychological distress in ethnic minority LGBTQA+ populations (Balsam et al., 2011). Further, LGBTQ+ based microaggressions within Aboriginal and Torres Strait Islander communities are associated with lower wellbeing among Aboriginal and Torres Strait Islander LGBTQ+ adults (Uink et al., 2024). Given these previous findings, we will assess whether microaggressions are a risk factor for low SEWB in the current sample. All examinations in Study 1 are exploratory given the novelty of the SEWB outcome. Study 2: Research Question. What are the risk and protective factors for mental health difficulties in Aboriginal and Torres Strait Islander LGBTQA+ young people? In this second set of analyses, we operationalise mental health difficulties in two ways: i) Psychological Distress and ii) Suicide Behaviour. The same putative protective factors discussed in Study 1 will be explored in relation to mental health difficulties. In addition, we set the following confirmatory hypotheses, based on prior research and theory (e.g. Intersectionality Theory, Minority Stress Theory): H1: There will be a negative relation between parental/caregiver acceptance and i) Psychological Distress and ii) Suicide Behaviour where higher parental/caregiver acceptance will be associated with lower Psychological Distress scores and lower likelihood of suicide behaviour within the last 12 months. Past research with non-Indigenous LGBTQ+ youth finds that parental acceptance is associated with better mental health outcomes (Fish, 2020), supporting our confirmatory approach. H2a: There will be a positive relation between number of race-based

microaggressions within the LGBTQA+ community and i) Psychological Distress scores and ii) likelihood of Suicide Behaviour within the last 12 months H2b: There will be a positive relation between level of distress associated with race-based microaggressions within the LGBTQA+ community and i) Psychological Distress and ii) likelihood of Suicide Behaviour within the last 12 months H3a: There will be a positive relation between number of LGBTQA+ microaggressions within Aboriginal and Torres Strait Islander communities and i) Psychological Distress scores and ii) likelihood of Suicide Behaviour within the last 12 months H3a: There will be a positive relation between level of distress due to LGBT+ microaggressions within Aboriginal and Torres Strait Islander communities and i) Psychological Distress scores and ii) likelihood of Suicide Behaviour within the last 12 months. H4a: There will be a positive relation between number of race-based microaggressions within romantic relationships and i) Psychological Distress scores and ii) likelihood of Suicide Behaviour within the last 12 months H4b: There will be a positive relation level of distress associated with race-based microaggressions within romantic relationships and i) Psychological Distress scores and ii) likelihood of Suicide Behaviour likelihood of Suicide Behaviour within the last 12 months. Hypotheses 2a-4b are set based on predictions of Intersectionality Theory and Minority Stress Theory. Additionally, we will test for interaction effects of each type of microaggression on i) Psychological Distress and ii) Likelihood of suicide behaviour within the last 12 months. Study 3: Does SEWB buffer the impacts of microaggressions on mental health difficulties? Although we originally treat participants SEWB score(s) as an outcome of interest (Study 1), for this set of analyses we turn our attention to the potential role of SEWB as a protective factor against discrimination experiences. We hypothesise that SEWB scores will moderate the relations between microaggressions and i) Psychological Distress scores and ii) Likelihood of Suicide Behaviour within the last 12 months such that the relation between microaggressions and mental health difficulties will be less strong. Examinations in Study 1 are exploratory given the novelty of the SEWB outcome among participants with higher SEWB.

**For interaction effects, describe the expected shape of the interactions.**

Minority Stress Theory (Meyer et al., 2003) proposes that among individuals with more than one marginalised identity, those who experience discrimination and stressors associated with both identities will experience poorer health outcomes compared to those who experience stressors associated with only one marginalised identity. To test this theory, we will assess: interactions between each type of microaggression measured in the survey (i.e., race-based microaggressions, LGBTQA+ microaggressions, romantic microaggressions) on SEWB outcomes (Study 1) and mental health difficulty outcomes (Study 2) to determine whether relations between microaggressions and SEWB and between microaggressions and mental health difficulties differ between participants who report higher microaggressions (both in terms of frequency and related distress) across multiple forms of microaggressions compared to participants who report only one type of microaggression. Buffering effect of SEWB: Study 3 H1: Participants with higher SEWB scores will show a weaker, positive association between each type of microaggression and i) Psychological Distress scores and ii) Likelihood of Suicide Behaviour within the last 12 months, compared to participants with lower SEWB scores.

**If you are manipulating a variable, make predictions for successful check variables or explain why no manipulation check is included.**

N/A as no variables will be manipulated in the study.

**Recommended elements**

**A figure or table may be helpful to describe complex interactions; this facilitates correct specification of the ordering of all group means.**

No files selected

**For original research, add rationales or theoretical frameworks for why a certain hypothesis is tested.**

Confirmatory hypotheses: Study 2: Parental acceptance for LGBTQA+ identity is a well established protective factor for LGBTQA+ youth mental health (Fish, 2020). Thus we expect that parent/caregiver acceptance will be associated with lower psychological distress and lower likelihood of suicide behaviour within the last 12 months in the current study. Prior research with POC LGBTQA+ populations has shown that microaggressions are associated with psychological distress (Balsam et al., 2011). In a previous study with Aboriginal and Torres Strait Islander LGBTQA+ adults (Uink et al., 2024), we found significant positive associations between LGBTQA+ based microaggressions in Aboriginal and Torres Strait Islander communities and wellbeing. Thus we expect that each type of microaggression will be associated with higher psychological distress and higher likelihood of suicidal behaviour within the last 12 months. Study 3: Components of SEWB including connection to Country, Culture, and Community have been

theorised to buffer the impacts of negative life experiences on Aboriginal and Torres Strait Islander youths' mental health outcomes (Salmon et al., 2018). Therefore we hypothesise that SEWB scores will moderate the relations between microaggressions and i) Psychological Distress scores and ii) Likelihood of Suicide Behaviour within the last 12 months such that the relation between microaggressions and mental health difficulties will be less strong among participants with higher SEWB. Exploratory analyses: All analyses assessing risk and protective factors for SEWB are exploratory. Risk and protective factors were drawn from qualitative work investigating the SEWB of Aboriginal and Torres Strait Islander LGBTQA+ young people (Liddelow-Hunt et al., 2021).

**If multiple predictions can be made for the same IV-DV combination, describe what outcome would be predicted by which theory.**

No data

**B. Methods - Essential elements**

**Description of essential elements**

**Design**

No data

**List, based on your hypotheses from section A: Independent variables with all their levels a. whether they are within- or between-participant b. the relationship between them (e.g., orthogonal, nested). Updated**

All variables are between-participant. i. *Positive Media Representation (3 items, "Aboriginal and Torres Strait Islander LGBTQA+ people are often represented fairly in the media", "I think it is important for Aboriginal and Torres Strait Islander LGBTQA+ people to be represented fairly in the media", "Seeing Aboriginal and Torres Strait Islander LGBTQA+ people represented fairly in the media makes me feel seen" [1 = Strongly disagree; 5 = Strongly Agree]). Average score will be used as final score. ii. Parent/Caregiver Acceptance (1 item, 'I feel that me being LGBTQA+ is accepted by the following people….my parent(s)/caregiver(s)' [1 = Strongly disagree; 4 = Strongly agree; 5 = Does not apply to me]). iii. *Sibling Acceptance (1 item, 'I feel that me being LGBTQA+ is accepted by the following people….sibling(s) [1 = Strongly disagree; 4 = Strongly agree; 5 = Does not apply to me]) iv. *Extended Family Acceptance (1 item, 'I feel that me being LGBTQA+ is accepted by the following people….members of my extended family [1 = Strongly disagree; 4 = Strongly agree; 5 = Does not apply to me]) v*. Elder acceptance (1 item 'I feel that me being LGBTQA+ is accepted by the following people: Aboriginal/Torres Strait Islander Elder(s) in my community: [1 = Strongly disagree; 4 = Strongly agree; 5 = Does not apply to me]) vii. *Community Acceptance (1 item, 'I feel that me being LGBTQA+ is accepted by the following people….my community [1 = Strongly disagree; 4 = Strongly agree; 5 = Does not apply to me]. viii. Identity Centrality (4 items, "Being Aboriginal and/or Torres Strait Islander LGBTQA+ is something that I don't think about much (reverse scored)", "For other people to know me properly, it is important for them to know that I am Aboriginal and/or Torres Strait Islander and LGBTQA+", "I don't have strong feelings about being Aboriginal and/or Torres Strait Islander and LGBTQA+" (reverse scored)", Being Aboriginal and/or Torres Strait Islander and LGBTQA+ is an important part of who I am." [1= Strongly disagree; 5 = Strongly agree] adapted from the Ethnic Centrality Subscale validated in LGBT Latinx youth (Fuligni et al., 2005; Shramko et al., 2018). Average score will be used as final score. ix). *Pride in Identity (2 items, "I am proud to be LGBTQA+.", "I am proud to be Aboriginal and/or Torres Strait Islander[1= Strongly disagree; 5 = Strongly agree]. Individual items will be used in analyses. x). Microaggressions (LGBT Racism 4 items, POC Heterosexism 4 items, LGBT Relationship Racism 2 items. Response options were yes/no. For participants who selected yes, a drop down question appeared asking 'how much did it bother you?' 0 = not at all; 4 = extremely. Items were adapted from Balsam et al., 2011 and Hill et al 2021. The microaggressions subscales will be scored in 2 ways: 1) Total score comprised on total number of microaggressions experienced within the last 12 months and 2) Severity score comprised of average severity rating for each microaggression reported (Balsam et al., 2014). *novel measures developed for current study.

**List dependent variables, or variables in a correlational design Updated**

SEWB variables (Study 1): i. Connection to Community (10 items: 'I feel I belong to…. an Aboriginal and Torres Strait Islander community; LGBTQA+ community; an Aboriginal and Torres Strait Islander LGBTQA+ community; youth community, 'other' community [1 = Strongly disagree; 5 = Strongly agree]; Frequency of participation in community events [0 = Never; 1 = Very often]). Items adapted from Berry et al., 2012. Belonging and Frequency scales will be treated as separate scales. Average scores on each scale will be used in all analyses. ii. Connection to

Country (2 items, ‘I feel like I belong to my people's land/waters’ [1 = Not at all 4 = A lot]; ‘Are you currently living off Country?’ (Yes, No, Unsure of Country), adapted from Berry et al., 2012. Items will be used individually. iii. Connection to Culture (2 items assessing participation in Cultural Activities; [0 = Never; 4 = Very often]). Adapted from Berry et al., 2012. Average scores will be used. iv. Connection to Family/Kinship (1 item assessing Family/Kinship Connection [1 = Strongly disagree; 5 = Strongly agree] adapted from Berry et al., 2012; 2 items assessing Family Functioning [1 = Strongly disagree; 5 = Strongly agree]. Connection and Family Functioning will be treated as separate. v. Connection to Spirit/Ancestors (1 item; “I have a strong sense of connection to my spirituality and ancestors” [0 = not at all true for me; 100 = completely true for me], adapted from Berry et al., 2012). Scores will be rescaled by dividing by 10. vi. Connection to Mind/Emotions (4 items adapted from the Inner Peace Subscale, Haswell et al. 2010 [0 = not at all true for me; 100 = completely true for me]). Scores will be rescaled by dividing by 10. Average scores will be used. vii. Connection to Body (1 item assessing self-rated overall physical health [0 = Extremely poor; 100 = Extremely good; 2 items assessing Substance Use ['do you use alcohol or drugs': 1 = No, never; 2 = Yes, monthly or less; 3 = Yes, 2-4 times a month; 4 = Yes, 2-3 times a week; 5 = Yes, 4 or more times a week; 'Has your life been made worse by your use of alcohol or drugs? 1 = No, not at all; 2 = No, not really; 3 = Neither yes or no; 4 = Yes, a bit; 5 = Yes a lot]. Physical Health Scores will be rescaled by dividing by 10. Substance Use items [use; impact] will be assessed individually. viii. Total SEWB - items from each SEWB sub-scale will be averaged to form a Total SEWB score if internal reliability is adequate. Mental Health Difficulties Variables (Study 2): i. Psychological Distress (Kessler 5 used to assess psychological symptoms within the past 4 weeks [1 = none of the time; 6 = all of the time] Bougie et al., 2016). Total score on the K5 will be used in all analyses. ii. Suicide Behaviour (4 items, ideation, making plan, suicide attempt within the last 12 months [y/no/prefer not to say), lifetime history of suicide attempt [y/n/prefer not to say] taken from Youth Risk Behavior Survey, Centre for Disease Control and Prevention, 2019) Each item will be treated as a separate outcome in all analyses. Tests of associations with microaggressions will not include lifetime history of suicide attempt as microaggressions are only reported over the last 12 months.

**Third variables acting as covariates or moderators.**

In study 3 each SEWB subscale (and Total SEWB score if appropriate) will be assessed as a moderator of the relations between each type of microaggression and each mental health difficulty outcomes. Demographic Variables: age (continuous), *gender, *trans status, *sexuality, cultural background (nominal variable, 0 = Aboriginal, 1 = Torres Strait Islander, 2 = Aboriginal and Torres Strait Islander); socioeconomic disadvantage (nominal variable based on participants’ postcode, 0 = low SES, 1 = medium SES, 2 = high SES ); urbanicity (nominal variable based on postcode data; 0 = urban; 1 = not-urban); parent/caregiver status (categorical variable, 0 = not parent/caregiver; 1 = parent/caregiver); disability status (categorical variable, 0 = no disability; 1 = disability); living situation (categorical variable, 0 = stable accommodation; 1 = insecure accommodation/experiencing homelessness). *variables will be coded once data is reviewed to ensure appropriate representation of the sample.

**Planned Sample**

No data

**If applicable, describe pre-selection rules.**

N/A

**Indicate where, from whom and how the data will be collected.**

This is a registration prior to analysis of the data. Data was collected for the purposes of the listed studies. Data were taken from a community sample of youth (14-25 years) who self-identified as Aboriginal and/or Torres Strait Islander and LGBTQA+ and who resided in Australia. Participants primarily completed the survey online which was hosted via Qualtrics software. Participants were recruited through social media, print flyers, e-newsletters for Aboriginal and Torres Strait Islander health and LGBTQA+ health, the project youth advisory and governance groups, and researcher professional networks. The study team also sent flyers and print copies of the survey to every Aboriginal Community Controlled Health Organisation (ACCHO) in Australia. Participants also had an option of calling the research team to complete the survey over-the-phone. As of the date of this pre-registration the data exist and have been accessed to determine rates of missing data and fidelity of responses. No other data analysis has been completed (including calculation of summary statistics).

**Justify planned sample size**

We determined desired sample size based on feasibility and statistical power for planned analyses. A recent survey with Aboriginal and Torres Strait Islander adults (18+) recruited approximately N = 60 participants from Western Australia (Hill et al., 2021). Using this prior sample as a guide, we estimated that we would recruit approximately 50 participants from each state and territory in Australia (total n = 350) which would be an appropriate sample size for planned regression analyses.

**If applicable, you can upload a file related to your power analysis here (e.g., a protocol of power analyses from G*Power, a script, a screenshot, etc.).**

No files selected

**Describe data collection termination rule.**

Data was collected from Feb 10th 2022 - July 31st 2022. We chose to keep the survey open until 1) our minimum sample size was reached and 2) survey response rate had trailed off and remained low for two weeks, indicating that no further responses would be attained.

**Exclusion Criteria**

No data

**Describe anticipated specific data exclusion criteria. For example: a) missing, erroneous, or overly consistent responses; b) failing check-tests or suspicion probes; c) demographic exclusions; d) data-based outlier criteria; e) method-based outlier criteria (e.g. too short or long response times).**

Exclusion criteria were i) not being able to give informed consent; ii) not being Aboriginal and/or Torres Strait Islander; iii) not being LGBTQA+ and iv) being younger than 14 years or older than 25 years. Data will be cleaned based on best-practice recommendations on identifying invalid data in online surveys (Curran, 2016) and include: • responses where participant meets any of the exclusion criteria • removal of clearly aberrant responses (e.g., hateful or joke comments given in open-ended questions; extremely fast completion time) • Removing cases with <5% completed data (i.e., 95% missing data)**

**Procedure**

No data

**Describe all manipulations, measures, materials and procedures including the order of presentation and the method of randomization and blinding (e.g., single or double blind), as in a published Methods section.**

N/A

**Recommended elements**

**Procedure**

No data

**Set fail-safe levels of exclusion at which the whole study needs to be stopped, altered, and restarted. You may pre-determine what proportion of excluded participants will cause the study to be stopped and restarted.**

N/A

**If applicable, you can upload any files related to your methods and procedure here (e.g., a paper describing a scale you are using, experimenter instructions, etc.)**

No files selected

**C. Analysis plan - Essential elements**

**Confirmatory Analyses**

**Describe the analyses that will test the first main prediction from the hypotheses section. Include:**

No data

**the relevant variables and how they are calculated; Updated**

H1-H4b: IVs - Parental/Caregiver acceptance (continuous variable), Race-Based micro-aggressions (continuous variable for frequency, continuous variable for related distress), LGBTQ+ micro aggressions (continuous variable for frequency, continuous variable for related distress), Relationship micro-aggressions (continuous variable for frequency, continuous variable for related distress). DVs - Psychological Distress (continuous score from K5), Suicidal ideation with last 12 months (dichotomous variable), Suicidal plan within last 12 months (dichotomous variable), Suicide attempt within last 12 months (dichotomous variable) Study 2 Exploratory analyses: IVs- demographic variables, positive media representation (continuous variable), sibling acceptance (continuous variable), extended family acceptance (continuous variable), elder acceptance (continuous variable), elder acceptance (continuous variable), community acceptance (continuous variable), identity centrality (continuous variable), pride in Aboriginal/Torres Strait Islander identity (continuous variable), pride in LGBTQA+ identity (continuous variable).

**the statistical technique;**

Univariate and multivariate regression for SEWB and K5 scores, logistic regression for suicide behaviour outcomes (suicidal ideation, suicide plan, suicide attempt within last 12 months). A series of univariate regression models will regress K5 (and then suicide behaviour scores) scores onto each demographic variable and each risk and protective factor. Factors that are significant at p <.10 will be included in final multiple regression models predicting K5/suicide behaviour. Factors with high multi-collinearity (r = >.80) will not be included in the multiple regression models. Model output will be assessed for model fit and per cent of variance explained by each factor.

**each variable's role in the technique (e.g., IV, DV, moderator, mediator, covariate); Updated**

Model 1-Parental/caregiver acceptance (IV), K5 scores (DV) Model 2- Race-based microaggressions in the LGBTQ+ community (IV), K5 scores (DV) Model 3-Race-based microaggressions in romantic relationships (IV), K5 scores (DV) Model 4- LBTQA+ based microaggressions within Aboriginal communities (IV), K5 scores (DV) Exploratory analyses: Model 5- Age (IV), K5 scores (DV) Model 6- Gender (IV), K5 scores (DV) Model 7- Trans status (IV), K5 scores (DV) Model 8- Sexuality (IV), K5 scores (DV) Model 9- Cultural background (IV), K5 scores (DV) Model 10- SES (IV), K5 scores (DV) Model 11- Urbanicity (IV) K5 scores (DV) Model 12- Parent/caregiver status (IV), K5 scores (DV) Model 13- Disability status (IV), K5 scores (DV) Model 14- Living situation (IV), K5 scores (DV) Model 15- Positive media representation (IV), K5 scores (DV) Model 16 - Sibling acceptance (IV), K5 scores (DV) Model 17- elder acceptance (IV), K5 scores (DV) Model 18- Extended family acceptance (IV), K5 scores (DV) Model 19- Elder acceptance (IV), K5 scores (DV) Model 20 - Community acceptance (IV), K5 scores (DV) Model 21- Identity centrality (IV), K5 scores (DV) Model 22- Pride in Aboriginal/Torres Strait Islander identity (IV), K5 scores (DV) Model 23- Pride in LGBTQA+ identity (IV), K5 scores (DV) *Model 24: Final multiple regression model with factors significant at p<.10 and <.80 -collinearity with other factors in included as IV's, K5 scores (DV). Models 1-21 will be run separately with each of the suicide behaviour outcomes (ideation, plan, attempt) using a logistic regression framework. Models 2-4 will be run separately with frequency of microaggressions and then with severity of microaggressions.

**rationale for each covariate used, if any;**

The demographic covariates were selected based on their likelihood of being associated with mental health difficulty outcomes.

**if using techniques other than null hypothesis testing (for example, Bayesian statistics), describe your criteria and inputs toward making an evidential conclusion, including prior values or distributions.**

N/A

**Second Prediction**

**Describe the analyses that will test the second main prediction from the hypotheses section. Include:**

No data

**the relevant variables and how they are calculated;**

Study 3: IVs- race-based microaggressions (continuous variable for frequency, continuous variable for related distress), LGBTQ+ microaggressions (continuous variable for frequency, continuous variable for related distress), dating microaggressions (continuous variable for frequency, continuous variable for related distress). Moderator variable: SEWB scores (continuous variable) DVs - Psychological Distress (continuous score from K5), Suicidal

ideation with last 12 months (dichotomous variable), Suicidal plan within last 12 months (dichotomous variable), Suicide attempt within last 12 months (dichotomous variable)

**the statistical technique;**

Logistic regression with moderator. Significant interaction effects will be probed by plotting simple slopes.

**each variable's role in the technique (e.g., IV, DV, moderator, mediator, covariate);**

Model 1: Race-based microaggressions* X SEWB score** (IV), K5 score (DV) Model 2: LGBTQA+ based microaggressions* x SEWB score (IV), K5 score (DV) Model 3: Dating microaggressions* x SEWB score (IV), K5 score (DV) Model 4: Race-based microaggressions x SEWB score (IV), Suicidal ideation (DV) Model 5: LGBTQ+ based microaggressions X SEWB score (IV), Suicidal ideation (DV) Model 6: Relationship microaggressions X SEWB score (IV), Suicidal ideation (DV) Model 7: Race-based microaggressions X SEWB score (IV), Suicide Plan (DV) Model 8: LGBTQ+ based microaggressions x SEWB score (IV), Suicide Plan (DV) Model 9: Relationship microaggressions X SEWB score (IV), Suicide Plan (DV) Model 10: Race-based microaggressions X SEWB score (IV), Suicide attempt in last 12 months (DV) Model 11: LGBTQ+ based microaggressions X SEWB score (IV), Suicide attempt in last 12 months (DV) Modell 12: dating microaggressions X SEWB score (IV), Suicide attempt in last 12 months (DV) *separate models will be run with microaggression frequency and severity. **individual SEWB domain scores will be used if there is low internal reliability for a Total SEWB scale.

**rationale for each covariate used, if any;**

Demographic co-variates found to be significant in Study 1 & 2 will be added to the models.

**if using techniques other than null hypothesis testing (for example, Bayesian statistics), describe your criteria and inputs toward making an evidential conclusion, including prior values or distributions.**

No data

**Third Prediction**

**Describe the analyses that will test the third main prediction from the hypotheses section. Include:**

No data

**the relevant variables and how they are calculated;**

No data

**the statistical technique;**

No data

**each variable's role in the technique (e.g., IV, DV, moderator, mediator, covariate);**

No data

**rationale for each covariate used, if any;**

No data

**if using techniques other than null hypothesis testing (for example, Bayesian statistics), describe your criteria and inputs toward making an evidential conclusion, including prior values or distributions.**

No data

**Fourth Prediction**

**Describe the analyses that will test the fourth main prediction from the hypotheses section. Include:**

No data

**the relevant variables and how they are calculated;**

No data

**the statistical technique;**

No data

**each variable’s role in the technique (e.g., IV, DV, moderator, mediator, covariate);**

No data

**rationale for each covariate used, if any;**

No data

**if using techniques other than null hypothesis testing (for example, Bayesian statistics), describe your criteria and inputs toward making an evidential conclusion, including prior values or distributions.**

No data

**Further Predictions**

**Describe the analyses that will test any further (main) predictions from the hypotheses section. Include:**

No data

**the relevant variables and how they are calculated;**

No data

**the statistical technique;**

No data

**each variable’s role in the technique (e.g., IV, DV, moderator, mediator, covariate);**

No data

**rationale for each covariate used, if any;**

No data

**if using techniques other than null hypothesis testing (for example, Bayesian statistics), describe your criteria and inputs toward making an evidential conclusion, including prior values or distributions.**

No data

**Recommended Elements**

**Specify contingencies and assumptions, such as:**

No data

**Method of correction for multiple tests.**

A Benjamini & Hochberg correction will be applied to applied to models with multiple comparisons.

**The method of missing data handling (e.g., pairwise or listwise deletion, imputation, interpolation).**

Missing data will be ignored listwise in simple descriptive analyses. Full information maximum likelihood in MPlus v8 will be used to handle missing data in regression models.

**Reliability criteria for item inclusion in scale.**

Items which require reverse scoring as per measure guidelines will be reverse scored. For scales with 2 items we will use Pearson’s correlation to assess internal consistency. Pearson’s r =>.70 will be considered evidence of acceptable internal reliability. For scales with 3 or more items we will use Cronbach’s alpha to measure internal reliability. Cronbach's alphas => .70 will be considered acceptable.

**Anticipated data transformations.**

Scripts for recoding demographic variables and for data scoring are available at https://archive.org/details/osf-registrations-dnj9p-v1.

**Assumptions of analyses, and plans for alternative/corrected analyses if each assumption is violated.**

IVs with multicollinearity >.80 will be excluded from multiple regression analyses. All regression analyses will be run in Mplus 8 using the MLR estimator to account for violations of normality. Outliers will be included in the analyses.

**Optionally, upload any files here that are related to your analyses (e.g., syntaxes, scripts, etc.).**

No files selected

**Final questions**

**Has data collection begun for this project?**

Yes, data collection is underway or complete

**If data collection has begun, have you looked at the data?**

Yes

**The (estimated) start and end dates for this project are**

July 2022-Dec 2024

**Any additional comments before I pre-register this project**

References:


Balsam, K. F., Molina, Y., Beadnell, B., Simoni, J., & Walters, K. (2011). Measuring multiple minority stress: the LGBT People of Color Microaggressions Scale. Cultural Diversity and Ethnic Minority Psychology, 17(2), 163.

Berry, S. L., Crowe, T. P., & Deane, F. P. (2012). Preliminary development and content validity of a measure of Australian Aboriginal cultural engagement. Ethnicity & health, 17(3), 325-336.

Bougie, E., Arim, R.G., Kohen, D.E., & Findlay, L.C. 2016. "Validation of the 10-item Kessler Psychological Distress Scale (K10) in the 2012 Aboriginal Peoples Survey." Health reports 27 (1): 3-10.

Centers for Disease Control and Prevention. Youth Risk Behavior Survey (YRBS) 2019 Standard Questionnaire Item Rationale. https://www.cdc.gov/yrbs/files/2019/pdf/2019_standard_yrbs_item_rationale.pdf (accessed 15 November 2025)

Crenshaw, K. (1991). Mapping the margins: Identity politics, intersectionality, and violence against women. Stanford Law Review, 43(6), 1241-1299.

Curran, P. G. (2016). Methods for the detection of carelessly invalid responses in survey data. Journal of Experimental Social Psychology, 66, 4-19.

Dudgeon, P., Bonson, D., Cox, A., Georgatos, G., & Rouhani, L. (2015). The Aboriginal and Torres Strait Islander suicide prevention evaluation project (ATSISPEP): Sexuality and gender diverse populations (lesbian, gay, bisexual, transsexual, queer and intersex—LGBTQI): Roundtable report. Canberra: The Healing Foundation.

Fish, J. N. (2020). Future directions in understanding and addressing mental health among LGBTQ youth. Journal of Clinical Child & Adolescent Psychology, 49(6), 943-956.

Fuligni AJ, Witkow M, Garcia C. Ethnic identity and the academic adjustment of adolescents from Mexican, Chinese, and European backgrounds. Developmental Psychology. 2005;41(5):799–811.

Gee, G., Dudgeon, P., Schultz, C., Hart, A., & Kelly, K. (2014). Aboriginal and Torres Strait Islander social and emotional wellbeing. Working together: Aboriginal and Torres Strait Islander mental health and wellbeing principles and practice, 2, 55-68.

Haswell, M. R., Kavanagh, D., Tsey, K., Reilly, L., Cadet-James, Y., Laliberte, A., ... & Doran, C. (2010). Psychometric validation of the Growth and Empowerment Measure (GEM) applied with Indigenous Australians. Australian & New Zealand Journal of Psychiatry, 44(9), 791-799.

Hill, B., Uink, B., Dodd, J., Bonson, D., Eades, A. M., & Bennett, S. (2021). Breaking the silence: Insights into the lived experiences of WA Aboriginal/LGBTIQ+ people: Community summary report 2021.

Liddelow-Hunt, S., Uink, B., Hill, B., Perry, Y., Munns, S., Talbott, T., Lin, A. (2021). Walkern Katatdjin (Rainbow Knowledge) Phase 1 Community Report, Perth, Western Australia.

Meyer, I. H. (2003). Prejudice, social stress, and mental health in lesbian, gay, and bisexual populations: conceptual issues and research evidence. Psychological bulletin, 129(5), 674.

Salmon, M., Doery, K., Dance, P., et al. Defining the indefinable: Descriptors of Aboriginal and Torres Strait Islander peoples' cultures and their links to health and wellbeing. (2018). The Lowitja Institute, Mayi Kuwayu. https://openresearch-repository.anu.edu.au/bitstreams/f3e9d39d-19fa-4f13-8e19-7d4e1dd48295/download (accessed Sept 2026).

Shramko, M., Toomey, R. B., & Anhalt, K. (2018). Profiles of minority stressors and identity centrality among sexual minority Latinx youth. American Journal of Orthopsychiatry, 88(4), 471.

Uink, B., Liddelow-Hunt, S., Daglas, K., & Ducasse, D. (2020). The time for inclusive care for Aboriginal and Torres Strait Islander LGBTQ+ young people is now. The Medical Journal of Australia, 213(5), 201-204.

Uink, Bep, Rebecca Bennett, Sian Bennett, Dameyon Bonson, and Braden Hill. "Considering First Nations LGBTIQ+ Identity in Anti-Racist Healthcare: Relations between Comfort in Healthcare, Microaggressions and Wellbeing." *First Nations Health and Wellbeing - The Lowitja Journal* 2 (2024). https://doi.org/10.1016/j.fnhli.2024.100027.

Additional notes:

**We relied on participant self-report of Aboriginal and Torres Strait Islander LGBTQA+ status which is the only method for verifying these identities in research. Scanning for extremely fast completion time or 'joke responses' further helped validate the survey respondents came from the population of interest. There were no financial incentives for completing the survey thus reducing the likelihood of fraudulent responses, including 'bot' responses.

**Table S1: How this study addresses the CONSIDER checklist (Huria et al., 2019)**

| | |
|---|---|
| **Governance** | |
| 1. Describe partnership agreements between the research institution and Indigenous-governing organization for the research, (e.g., Informal agreements through to MOU (Memorandum of Understanding) or MOA (Memorandum of Agreement)). | The partnership with the project's Youth Advisory Group and Governance Committee follows a Research Collaboration Agreement that describes the roles and expectations of all parties. Relationships with Indigenous partner organisation were formalised either through a Research Collaboration Agreement created by the research institution or an agreement created by the Indigenous organisation, depending on the organisation's preference and existing processes for research review. Community members and researchers reviewed and negotiated the agreements to ensure that free prior informed consent was in place for all research partnerships. |
| 2. Describe accountability and review mechanisms within the partnership agreement that addresses harm minimization. | The research team regularly report on progress and future plans to the Youth Advisory Group and Governance Committee during quarterly meetings. The Research Collaboration Agreements or similar with the Youth Advisory Group, Governance Committee and partner organisations detail fair processes for conflict resolution. All participants and partners are provided with the details of the approving Aboriginal health ethics committee in their jurisdiction so that there is a clear process for complaints. |
| 3. Specify how the research partnership agreement includes protection of Indigenous intellectual property and knowledge arising from the research, including financial and intellectual benefits generated (e.g., development of traditional medicines for commercial purposes or supporting the Indigenous community to develop commercialization proposals generated from the research). | Indigenous intellectual property and knowledge arising from the research is protected by the project's Indigenous Data Sovereignty Statement, which is publicly available on the study website. All decisions about the use of project data are made by the Youth Advisory Group and Governance Committee. All members of the Youth Advisory Group and Governance Committee were invited to take part in writing this manuscript and offered co-authorship to ensure they receive fair recognition for their intellectual contributions to the project, provided they met the journal's minimum requirements for authorship. Three members of the Youth Advisory Group are co-authors of this paper. |
| **Prioritization** | |
| 4. Explain how the research aims emerged from priorities identified by either Indigenous stakeholders, governing bodies, funders, non-government organization(s), stakeholders, consumers, and empirical evidence | This research emerged in direct response to calls from community organisations, advocates and leaders for evidence about the mental health of Aboriginal and Torres Strait Islander LGBTQA+ young people. This was confirmed by local community leaders and Elders while the research idea was being developed. The research team has remained responsive to the needs and priorities expressed by community throughout the project. This has been done through further data analysis, developing targeted resources, sharing findings with community in appropriate ways, and developing further research projects. |
| **Relationships (Indigenous stakeholders/participants and Research team)** | |
| 5. Specify measures that adhere and honour Indigenous ethical guidelines, processes, and approvals for all relevant Indigenous stakeholders, recognizing that multiple Indigenous partners may be involved, e.g., Indigenous ethics committee approval, regional/national ethics approval processes. | This research project received ethics approval from the Aboriginal health ethics committee in each jurisdiction across Australia and follows the *National Health and Medical Research Council (NHMRC) Ethical conduct in research with Aboriginal and Torres Strait Islander peoples and communities: Guidelines for researchers and stakeholders 2018*. As such, the project has also received written endorsement from multiple Aboriginal Community Controlled Organisations across Australia, most of which have their own processes for research review and approval. |
| 6. Report how Indigenous stakeholders were involved in the research processes (i.e., research | 3 of the 5 chief investigators on the project grant application are Aboriginal people. The project is led by Prof Uink, a Noongar woman. All aspects of the research project were developed in |

| | |
|---|---|
| design, funding, implementation, analysis, dissemination/recruitment). | partnership with the Youth Advisory Group and Governance Committee. For example, the coding framework for gender and sexuality used in this analysis was developed during a coding session with the Youth Advisory Group. The Youth Advisory Group and Governance Committee also provided extensive feedback on the draft survey, input into recruitment methods, assisted with interpreting the study findings, reviewed and approved this manuscript, and advised on further avenues to translate the research findings into action. Additionally, the research project has developed a network of Aboriginal and Torres Strait Islander LGBTQA+ people across Australia, and those relationships help to ensure that the research is meeting communities' data needs. |
| 7. Describe the expertise of the research team in Indigenous health and research. | The 1st author is a heterosexual, cisgender Noongar woman with experience in working on other research projects as well as specifically on Aboriginal LGBTQA+ projects. They work with local Elders and Aboriginal Community Controlled Organisations.<br>The 2nd author is a queer Yamatji person with a PhD in Population and Global Health. They have worked across a number of research projects concerning Aboriginal and Torres Strait Islander LGBTQA+ peoples' wellbeing and actively contribute to developing local processes for Indigenous Data Sovereignty. The have previously worked with Indigenous young people as a tutor and mentor.<br>The 3rd author is a non-Indigenous person who works with Aboriginal communities and data to progress data governance and streamlining of national datasets.<br>Authors 4 and 5 are members of the project Youth Advisory Group and have diverse experience in working with their communities to strengthen health outcomes.<br>Authors 6 and 7 are queer Noongar people who have extensive experience in Aboriginal community health research.<br>Authors 8 and 9 are non-Indigenous researchers who have developed relationships with LGBTQA+ and/or Aboriginal communities which have informed this research study. Through those projects they have developed relationships with Aboriginal communities that have informed this research project as well as gave expertise on ethics, methods and reporting. |
| **Methodologies** | |
| 8. Describe the methodological approach of the research including a rationale of methods used and implication for Indigenous stakeholders, e.g., privacy and confidentiality (individual and collective). | This research is situated within a critical understanding that settler colonialism has reshaped understandings of gender and sexuality to the detriment of Aboriginal and Torres Strait Islander LGBTQA+ people's wellbeing. By bringing together Aboriginal and Torres Strait Islander LGBTQA+ people through the governance and broader work of the project, the research has provided an opportunity to discuss and further understand how diversity can be expressed and embraced within Aboriginal and Torres Strait Islander Culture.<br>As a primarily online survey, participation required approximately year 9 English reading comprehension. To increase accessibility, participants were also able to complete the survey verbally over the phone with a member of the research team, complete the survey with a support person, or contact the research team to request the survey be translated into an Indigenous language. |
| 9. Describe how the research methodology incorporated consideration of the physical, social, economic and cultural environment of the participants and prospective participants. (e.g., | This analysis was derived from a qualitative research project in which Aboriginal LGBTQA+ young people identified the factors they see as contributing to their mental health and wellbeing. The variables selected for analysis include |

| | |
|---|---|
| impacts of colonization, racism, and social justice) as well as Indigenous worldviews. | acceptance by family, community and Elders, noting their role within an Aboriginal social and emotional wellbeing framework. It also includes measures of racism within the LGBTQA+ community and racism in romantic relationships. Identifying strengths-based factors that reduce the odds of poor wellbeing for Aboriginal and Torres Strait Islander LGBTQA+ young people provides evidence that can be leveraged for community-led responses. |
| **Participation** | |
| 10. Specify how individual and collective consent was sought to conduct future analysis on collected samples and data (e.g., additional secondary analyses; third-parties accessing samples (genetic, tissue, blood) for further analyses). | All decisions about further analysis of participant data, including by external parties, are made by the Youth Advisory Group and Governance Committee. The data request process established helps to ensure that the data can be used sustainably to provide much-needed evidence and reduce the research burden on community, but that data is only used in the best interests of the Aboriginal and Torres Strait Islander LGBTQA+ community and is protective of individual participants. Participants cannot be re-contacted because the survey was anonymous. |
| 11. Described how the resource demands (current and future) placed on Indigenous participants and communities involved in the research were identified and agreed upon including any resourcing for participation, knowledge, and expertise. | The Research Collaboration Agreements with the Youth Advisory Group, Governance Committee and partner organisations (as above) detail expectations of all parties including expected time commitment, expertise and access to other resources (e.g. sharing recruitment materials). The participation of the Youth Advisory Group and Governance Committee has been flexible throughout the project, with the understanding that community members having competing priorities that at times impact their capacity to contribute to research. Youth Advisory Group and Governance Committee members are reimbursed for their time if contributing to the research outside of the responsibilities of a paid role. |
| 12. Specify how biological tissue and other samples including data were stored, explaining the processes of removal from traditional lands, if done, and of disposal. | N/A. No biological samples were collected. |
| **Capacity** | |
| 13. Explain how the research supported the development and maintenance of Indigenous research capacity (e.g., specific funding of Indigenous researchers). | The 2nd author is a Wajarri person who completed a PhD through this research project. The 7th author is a queer Noongar person previously employed on this research project at 0.6 FTE and is now employed on a related project led by the 1st author. The 1st author was employed as a 0.4FTE Research Fellow at the commencement of this study and has since moved to another role with dedicated research FTE. Authors 1, 6 and 7 are Noongar researchers who have received research mentorship from other members of the research team. Reciprocally, non-Indigenous researchers (author 3, 8, 9) have received mentorship from Aboriginal investigators. Authors 4 and 5 are Aboriginal people engaged through the Youth Advisory Group who have receive opportunities for authorship, professional development, and co-presenting. The research team has sought to develop the research capacity of Indigenous students through supervising multiple Indigenous interns. The research team has also developed the data governance capacity of local Elders through engagement on multiple projects. |
| 14. Discuss how the research team undertook professional development opportunities to develop the capacity to partner with Indigenous stakeholders? | Development for non-Indigenous researchers occurs informally through working with the Aboriginal researchers who lead the research project. |
| **Analysis and interpretation** | |

| | |
|---|---|
| 15. Specify how the research analysis and reporting supported critical inquiry and a strength-based approach that was inclusive of Indigenous values. | This manuscript reports on an analysis of factors contributing to wellbeing for Aboriginal and Torres Strait Islander LGBTQA+ young people including family acceptance, queerphobia, and racism. As such, we explicitly seek to explore how disproportionate rates of poor mental health in this population are the result of structural oppression, not inherent vulnerability. |
| **Dissemination** | |
| 16. Describe the dissemination of the research findings to relevant Indigenous governing bodies and peoples. | The research findings have been shared through yarns with local Elders, presentations to clinical and professional networks, publicly accessible webinars for community, presentations at conferences. Additionally findings have been incorporated into resources for community. |
| 17. Discuss the process for knowledge translation and implementation to support Indigenous advancement (e.g., research capacity, policy, investment). | The findings of this survey have been used with and by Indigenous communities to advocate for Aboriginal LGBTQA+ support in services, policy, and community. Following the national survey, the research team co-designed a Roadmap with Indigenous community that articulates a community-led response to ensuring Aboriginal and Torres Strait Islander LGBTQA+ young people are safe, healthy and thriving. This includes specific actions that the research team have completed or advocated for in their broader professional roles, for example through providing input on the development on inclusion training or advocating for community priorities in state and federal government strategy.<br>Findings from the study have also informed the development of an inclusive practice training program for Aboriginal Community Controlled Organisations as well as a pilot study on an intergenerational support program bringing together Aboriginal LGBTQA+ young people, Elders, and community members to discuss the intersection of gender, sexuality and culture. |

**Table S2: STROBE Statement—Checklist of items that should be included in reports of cross-sectional studies**

| | Item No | Recommendation | Page number |
|---|---|---|---|
| **Title and abstract** | 1 | (*a*) Indicate the study's design with a commonly used term in the title or the abstract | 1 |
| | | (*b*) Provide in the abstract an informative and balanced summary of what was done and what was found | 1 |
| **Introduction** | | | |
| Background/rationale | 2 | Explain the scientific background and rationale for the investigation being reported | 3 |
| Objectives | 3 | State specific objectives, including any prespecified hypotheses | 4 |
| **Methods** | | | |
| Study design | 4 | Present key elements of study design early in the paper | 4 |
| Setting | 5 | Describe the setting, locations, and relevant dates, including periods of recruitment, exposure, follow-up, and data collection | 4 |
| Participants | 6 | (*a*) Give the eligibility criteria, and the sources and methods of selection of participants | 5 |
| Variables | 7 | Clearly define all outcomes, exposures, predictors, potential confounders, and effect modifiers. Give diagnostic criteria, if applicable | 5 |
| Data sources/ measurement | 8* | For each variable of interest, give sources of data and details of methods of assessment (measurement). Describe comparability of assessment methods if there is more than one group | 5 |
| Bias | 9 | Describe any efforts to address potential sources of bias | 5,8 |
| Study size | 10 | Explain how the study size was arrived at | 8 |
| Quantitative variables | 11 | Explain how quantitative variables were handled in the analyses. If applicable, describe which groupings were chosen and why | 5 |
| Statistical methods | 12 | (*a*) Describe all statistical methods, including those used to control for confounding | 8 |
| | | (*b*) Describe any methods used to examine subgroups and interactions | 9 |
| | | (*c*) Explain how missing data were addressed | 9 |
| | | (*d*) If applicable, describe analytical methods taking account of sampling strategy | 8 |
| | | (*e*) Describe any sensitivity analyses | 9 |
| **Results** | | | |
| Participants | 13* | (a) Report numbers of individuals at each stage of study—eg numbers potentially eligible, examined for eligibility, confirmed eligible, included in the study, completing follow-up, and analysed | 9 |
| | | (b) Give reasons for non-participation at each stage | N/A |
| | | (c) Consider use of a flow diagram | N/A |
| Descriptive data | 14* | (a) Give characteristics of study participants (eg demographic, clinical, social) and information on exposures and potential confounders | 9 |
| | | (b) Indicate number of participants with missing data for each variable of interest | T1 |
| Outcome data | 15* | Report numbers of outcome events or summary measures | 10 |
| Main results | 16 | (*a*) Give unadjusted estimates and, if applicable, confounder-adjusted estimates and their precision (eg, 95% confidence interval). Make clear which confounders were adjusted for and why they were included | T2-4 |
| | | (*b*) Report category boundaries when continuous variables were categorized | N/A |

| | | | |
|---|---|---|---|
| | | (*c*) If relevant, consider translating estimates of relative risk into absolute risk for a meaningful time period | N/A |
| Other analyses | 17 | Report other analyses done—eg analyses of subgroups and interactions, and sensitivity analyses | N/A |
| **Discussion** | | | |
| Key results | 18 | Summarise key results with reference to study objectives | 12 |
| Limitations | 19 | Discuss limitations of the study, taking into account sources of potential bias or imprecision. Discuss both direction and magnitude of any potential bias | 13 |
| Interpretation | 20 | Give a cautious overall interpretation of results considering objectives, limitations, multiplicity of analyses, results from similar studies, and other relevant evidence | 14 |
| Generalisability | 21 | Discuss the generalisability (external validity) of the study results | 13 |
| **Other information** | | | |
| Funding | 22 | Give the source of funding and the role of the funders for the present study and, if applicable, for the original study on which the present article is based | 14 |

*Give information separately for exposed and unexposed groups.

**Note:** An Explanation and Elaboration article discusses each checklist item and gives methodological background and published examples of transparent reporting. The STROBE checklist is best used in conjunction with this article (freely available on the Web sites of PLoS Medicine at http://www.plosmedicine.org/, Annals of Internal Medicine at http://www.annals.org/, and Epidemiology at http://www.epidem.com/). Information on the STROBE Initiative is available at www.strobe-statement.org.

**Table S3: Record of deviations from pre-registration. Record format based on Willroth & Atherton, 2024.**

| No. | | Details | Original wording | Deviation description | Reader impact |
|---|---|---|---|---|---|
| 1 | Type | Covariates: Removed covariates | Demographic Variables: age (continuous), *gender, *trans status, *sexuality, **cultural background (nominal variable, 0 = Aboriginal, 1 = Torres Strait Islander, 2 = Aboriginal and Torres Strait Islander)**; **socioeconomic disadvantage (nominal variable based on participants' postcode, 0 = low SES, 1 = medium SES, 2 = high SES ); urbanicity (nominal variable based on postcode data; 0 = urban; 1 = not-urban); parent/caregiver status (categorical variable, 0 = not parent/caregiver; 1 = parent/caregiver)**; disability status (categorical variable, 0 = no disability; 1 = disability); **living situation (categorical variable, 0 = stable accommodation; 1 = insecure accommodation/experiencing homelessness)**. *variables will be coded once data is reviewed to ensure appropriate representation of the sample. | Cultural background, caregiver status, and stable accommodation removed as categorical demographic predictors due to small number of 'yes' responses. Socioeconomic disadvantage and urbanicity removed because they cannot be accurately determined using postcode data from the 2020 Australian census. | **Minor.** Demographic predictor variables that are potentially correlated with wellbeing will no longer be included, meaning we cannot determine if they are relevant to this cohort. |
| | Reason | Preregistered plan not possible or inappropriate because of data: Cell sizes are too small to analyse categorical variables as planned | | | |
| | Timing | After data access | | | |
| 2 | Type | Covariates: Used a different operationalisation of a covariate | Demographic Variables: → updated Oct 7 2022: Change in coding. | For gender diversity variable: 'unsure' and 'prefer not to say' categories were combined. For gender variable: non-binary, another gender and questioning were combined. For sexuality variable: asexual, queer, questioning, and another sexuality were combined. | Minimal. Loss in the ability to distinguish between some participants' diverse experiences of gender, gender diversity, and sexuality. |
| | Reason | Preregistered plan not possible or inappropriate because of data: Cell sizes are too small to analyse categorical variables as planned | | | |
| | Timing | After data access | | | |
| 3 | Type | Inferential criteria: Changed criteria for variable exclusion in multivariate models | [In relation to related study (Study 2)]: A series of univariate regression models will regress K5 (and then suicide behaviour scores) scores onto each demographic variable and each risk and | Peer review feedback and additional consultation with biostatistician indicated that using p <.10 as a cut-off for determination of | **Minor**. Allows reader greater confidence in study findings as analytical methods align with current best practice. |

| | | | | | |
|---|---|---|---|---|---|
| | Reason | Researchers gained new knowledge: The researchers discovered an existing analytic approach they were not previously aware of/One or more coauthors was added to the project after preregistering and offers new expertise or conflicting opinions about the best analytic approach | protective factor. Factors that are significant at p <.10 will be included in final multiple regression models predicting K5/suicide behaviour. Factors with high multi-collinearity (r = >.80) will not be included in the multiple regression models. Model output will be assessed for model fit and per cent of variance explained by each factor.<br>Note: same approach to variable inclusion was used in Study 1. | variable inclusion in multivariate models is no longer considered best practice; significance testing not appropriate for study aim (description of relationships between study variables). New exclusion criteria for variables in multivariate models were based on 1) high correlations with other exposure variables (r >=.70), or 2) conceptual/theoretical relevance; or 3) non-linear relationship with outcome; or 4) high positive or negative skew with inspection of histograms suggesting low variability; or 5) visual inspection of scattergrams/boxplots suggesting no relationship. | |
| | Timing | After data access | | | |
| 4 | Type | Variable operationalization or computation: Recoded variables | Microaggressions (LGBT Racism 4 items, POC Heterosexism 4 items, LGBT Relationship Racism 2 items. Response options were yes/no. For participants who selected yes, a drop-down question appeared asking 'how much did it bother you?' 0 = not at all; 4 = extremely. Items were adapted from Balsam et al., 2011 and Hill et al 2021.<br>The microaggressions subscales will be scored in 2 ways: 1) Total score comprised on total number of microaggressions experienced within the last 12 months and 2) Severity score comprised of average severity rating for each microaggression reported (Balsam et al., 2011). Interactions between each type of microaggression measured in the survey (i.e., race-based microaggressions, LGBTQA+ microaggressions, romantic | Microaggression frequency and microaggression severity (impact) were recoded into one composite 'frequency and impact' score for each subscale, due to small number of responses to branched 'impact' questions drastically reducing sample size. Both separate and composite scales were suggested by the original authors of the measure and found to produce similar results. Scale now (0) happened, (1) happened but didn't bother me, (2) happened and bothered me a little, (3) happened and bothered me a bit, (4) | **Minimal**. Choice of composite scale is within the parameters of the measure design and was found to be equally valid (Balsam et al., 2011). |
| | Reason | Preregistered plan not possible or inappropriate because of data: Extreme restriction of range for a continuous variable | | | |
| | Timing | After data access | | | |

| | | | | | |
|---|---|---|---|---|---|
| | | | microaggressions) on SEWB outcomes (Study 1). | happened and bother me quite a bit, (5) happened and bothered me a lot. Interaction effects removed due to composite scoring. | |
| 5 | Type | Variable operationalization or computation: Removed indicators | Positive Media Representation (3 items, “Aboriginal and Torres Strait Islander LGBTQA+ people are often represented fairly in the media”, “I think it is important for Aboriginal and Torres Strait Islander LGBTQA+ people to be represented fairly in the media”, “Seeing Aboriginal and Torres Strait Islander LGBTQA+ people represented fairly in the media makes me feel seen” [1 = Strongly disagree; 5 = Strongly Agree]). Average score will be used as final score. | Individual item “Aboriginal and Torres Strait Islander LGBTQA+ people are often represented fairly in the media” used in models due to high skew in items “I think it is important for Aboriginal and Torres Strait Islander LGBTQA+ people to be represented fairly in the media” and “Seeing Aboriginal and Torres Strait Islander LGBTQA+ people represented fairly in the media makes me feel seen” (small cell sizes for strongly disagree, disagree, and neutral). | **Minimal**. Assumes that perception of fair media representation has a stronger relationship with wellbeing outcomes than importance of fair representation and feeling seen by fair representation. |
| | Reason | Preregistered plan not possible or inappropriate because of data: Extreme restriction of range for a continuous variable | | | |
| | Timing | After data access | | | |
| 6 | Type | Variable operationalization or computation: Combined indicators | ii. Parent/Caregiver Acceptance (1 item, ‘I feel that me being LGBTQA+ is accepted by the following people….my parent(s)/caregiver(s)’ [1 = Strongly disagree; 4 = Strongly agree; 5 = Does not apply to me]). iii. *Sibling Acceptance (1 item, ‘I feel that me being LGBTQA+ is accepted by the following people….sibling(s) [1 = Strongly disagree; 4 = Strongly agree; 5 = Does not apply to me]) iv. *Extended Family Acceptance (1 item, ‘I feel that me being LGBTQA+ is accepted by the following people….members of my extended family [1 = Strongly disagree; 4 = Strongly agree; 5 = Does not apply to me]) v*. Elder acceptance (1 item 'I feel that me being LGBTQA+ is accepted by the following people: Aboriginal/Torres Strait Islander Elder(s) in my community: [1 = Strongly | Used an ‘acceptance’ variable computed from the average of parent/caregiver acceptance, sibling acceptance, extended family acceptance, Elder acceptance and community acceptance. Acceptance average variable will consider the impact of the overall acceptance young people receive, rather than support acceptance individual sources. | **Minimal.** Assumes that overall levels of acceptance are more influential on wellbeing outcomes health than acceptance from individual sources, which may be disputed. |
| | Reason | Preregistered plan not possible or inappropriate because of data: Extreme restriction of range for a continuous variable | | | |
| | Timing | After data access | | | |

| | | | | | |
|---|---|---|---|---|---|
| | | | disagree; 4 = Strongly agree; 5 = Does not apply to me]) vii. *Community Acceptance (1 item, ‘I feel that me being LGBTQA+ is accepted by the following people….my community [1 = Strongly disagree; 4 = Strongly agree; 5 = Does not apply to me]. | | |
| 7 | Type | Variable operationalization or computation: Removal of three outcomes variables. | ii. Connection to Country (2 items, ‘I feel like I belong to my people's land/waters’ [1 = Not at all 4 = A lot]; ‘Are you currently living off Country?’ (Yes, No, Unsure of Country), adapted from Berry et al., 2012. Items will be used individually.<br>vii. Connection to Body (1 item assessing self-rated overall physical health [0 = Extremely poor; 100 = Extremely good, Galan et al., 2013]; 2 items assessing Substance Use ['do you use alcohol or drugs': 1 = No, never; 2 = Yes, monthly or less; 3 = Yes, 2-4 times a month; 4 = Yes, 2-3 times a week; 5 = Yes, 4 or more times a week; 'Has your life been made worse by your use of alcohol or drugs? 1 = No, not at all; 2 = No, not really; 3 = Neither yes or no; 4 = Yes, a bit; 5 = Yes a lot]. Physical Health Scores will be rescaled by dividing by 10. Substance Use items [use; impact] will be assessed individually.<br>iv. Connection to Family/Kinship (1 item assessing Family/Kinship Connection [1 = Strongly disagree; 5 = Strongly agree] adapted from Berry et al., 2012; 2 items assessing Family Functioning [1 = Strongly disagree; 5 = Strongly agree] taken from Carlisle et al., 2019). Connection and Family Functioning will be treated as separate. | ‘Are you currently living off Country” and Family Functioning removed as outcomes due to external factors independent from Aboriginal LGBTQA+ youth status likely influencing them. Substance Use removed as outcome as Substance Use Impact deemed most critical for wellbeing. | **Minor.** Allows readers to focus on indicators which are unique to the population of interest. |
| | Reason | Researchers gained new knowledge: greater conceptual alignment with the SEWB model | | | |
| | Timing | After data access | | | |
| 8 | Type | Variable operationalization or computation: Addition of two outcome variables | Specifically, we will explore the relations between participants' SEWB scores and following putative protective factors ….viii) pride in Aboriginal and Torres Strait | Pride in Aboriginal and Torres Strait Islander identity and pride in LGBTQA+ identity became additional measures | **Minor.** Provides readers access to additional measures of Connection to Mind and Emotions domain. |

| | | | | | |
|---|---|---|---|---|---|
| | Reason | Researchers gained new knowledge: greater conceptual alignment with the SEWB model | Islander identity; ix) pride in LGBTQA+ identity. | of the Connection to Mind and Emotions domain to more comprehensively capture this SEWB domain. | |
| | Timing | After data access | | | |

**Table S4. Full list of study exposure variable items and wellbeing outcome items.**

| Question stem | Question |
|---|---|
| ***Exposure variables*** | |
| *Fair media representation* | |
| Please show how much you agree or disagree by sliding the marker along the scale. | Aboriginal and Torres Strait Islander LGBTQA+ people are often represented fairly in the media. |
| *Acceptance of LGBTQA+ identity* | |
| I feel that me being LGBTQA+ is accepted by the following people: | My Parent(s)/caregiver(s) |
| | My Sibling(s) |
| | My extended family (e.g. cousins, aunties, nephews): |
| | My community |
| | Aboriginal/Torres Strait Islander Elder(s) in my community |
| *LGBTQA+ racism* | |
| Please tick whether each of the following events have happened to you within the last 12 months. | Feeling like non-Indigenous LGBTQA+ people do not understand you.<br>IF YES → When you felt like non-Indigenous LGBTQA+ people did not understand you, how much did it bother you? |
| | Having to educate non-Indigenous LGBTQA+ people about race/cultural issues.<br>IF YES→ When you had to educate non-Indigenous LGBTQA+ people about race/cultural issues, how much did it bother you? |
| | Being the "token" Aboriginal and/or Torres Strait Islander LGBTQA+ person in a group or organization<br>IF YES → When you were the "token" Aboriginal and/or Torres Strait Islander LGBTQA+ person in a group or organisation, how much did it bother you? |
| | Non-Indigenous LGBTQA+ people saying things that are racist.<br>IF YES → When non-Indigenous LGBTQA+ people said things that were racist, how much did it bother you? |
| *Aboriginal and Torres Strait Islander queerphobia* | |
| Please tick each of the following events have happened to you within the last 12 months.<br>. | Feeling like people in the Aboriginal and Torres Strait Islander community do not understand you.<br>IF YES → When you felt like people in the Aboriginal and Torres Strait Islander community did not understand you, how much did it bother you? |
| | Feeling like your LGBTQA+ identity is not seen in the Aboriginal and Torres Strait Islander community.<br>IF YES → When you felt like your LGBTQA+ identity was not seen in the Aboriginal and Torres Strait Islander community, how much did it bother you? |
| | Having a hard time finding friends who are Aboriginal and/or Torres Strait Islander and LGBTQA+.<br>IF YES → When you had a hard time finding friends who were Aboriginal and/or Torres Strait Islander and LGBTQA+, how much did it bother you? |
| | Not having any Aboriginal and/or Torres Strait Islander LGBTQA+ people as role models.<br>IF YES → When you did not have any Aboriginal and/or Torres Strait Islander LGBTQA+ people as role models, how much did it bother you? |
| *Relationship racism* | |
| Please tick whether each of the following events have happened to you within the last 12 months. | Feeling like non-Indigenous LGBTQA+ people are only interested in you because of the way you look.<br>IF YES →<br>When you felt like non-Indigenous LGBTQA+ people were only interested in you because of the way you look, how much did it bother you? |
| | Being rejected by people you want to date or have sex with because you are Aboriginal and/or Torres Strait Islander.<br>IF YES →<br>When you were rejected by people you wanted to date or have sex with because you are Aboriginal and/or Torres Strait Islander, how much did it bother you? |
| ***Connection to Mind & Emotions*** | |

| | |
|---|---|
| *Inner Peace* | |
| From 1-100 how true are these? Move the marker to show your answer. | I have confidence in myself. |
| | I feel very happy with myself and my life. |
| | I feel safe and secure and as if I can face whatever is ahead. |
| | I can speak out and explain my views and people listen. |
| *Pride in LGBTQA+ identity* | |
| Please show us how much you agree or disagree with the following statements: | I am proud to be LGBTQA+ |
| *Pride in Aboriginal and Torres Strait Islander identity* | |
| Please show us how much you agree or disagree with the following statements: | I am proud to be Aboriginal and/or Torres Strait Islander. |
| ***Connection to Family and Kinships*** | |
| This is about being Aboriginal and/or Torres Strait Islander and LGBTQA+ with your family, friends and community. Please answer as honestly as possible. | I have a strong sense of kinship/family links. |
| ***Connection to Culture*** | |
| This is about culture. People celebrate their culture in different ways. These are just some examples. Please tell us how often you: | Take part in Aboriginal and/or Torres Strait Islander cultural practices (e.g. traditional or modern music/dance, food preparation, smoking ceremonies). |
| | Spend time trying to learn about Aboriginal and/or Torres Strait Islander culture, such as its history, traditions, language and customs. |
| ***Connection to Spirit, Spirituality and Ancestors*** | |
| From 1-100 how true are these? Move the marker to show your answer. | I have a strong sense of connection to my spirituality and ancestors. |
| ***Connection to Country*** | |
| Some Aboriginal and Torres Strait Islander people are very connected to Country, Culture and Community, and others aren't - often for reasons they can't control. Either way, it's okay. This is not a test of "how Aboriginal you are." We just want to see if connection is good for your wellbeing. | I feel like I belong to my people's land/waters. |
| ***Connection to Community*** | |
| *Belonging LGBTQA+ community* | |
| I feel I belong to: | …an LGBTQA+ community |
| *Belonging Aboriginal and Torres Strait Islander community* | |
| I feel I belong to: | …....an Aboriginal and/or Torres Strait Islander community. |
| *Belonging Aboriginal and Torres Strait Islander LGBTQA+ community* | |
| I feel I belong to: | ...an Aboriginal and/or Torres Strait Islander and LGBTQA+ community. |
| *Participation in LGBTQA+ community events* | |

| | |
|---|---|
| How often do you take part in: | Events or activities in an LGBTQA+ community (e.g. Pride Parade, joining an online LGBTQA+ group). |
| *Participation in Aboriginal and Torres Strait Islander community events* | |
| How often do you take part in: | Events or activities in the Aboriginal and/or Torres Strait Islander community (e.g. celebrating NAIDOC Week, attending community meetings). |
| *Participation in Aboriginal and Torres Strait Islander LGBTQA+ community events* | |
| How often do you take part in: | Events or activities in the Aboriginal and/or Torres Strait Islander LGBTQA+ community. |
| ***Connection to Body*** | |
| *Physical Health* | |
| This is about your physical and emotional health. | How is your overall physical health? |
| *Substance use* | |
| The next few questions are about alcohol and drug use among you and your family. We acknowledge that lots of Aboriginal and Torres Strait Islander people never use alcohol and drugs, and that it is often a negative stereotype of our families. Some Aboriginal and Torres Strait Islander people use alcohol or drugs and it does not cause them any problems. Other people find that their use of alcohol or drugs does cause them problems. We are interested to find out if this has happened to your or a family member. | Do you use alcohol or drugs?<br>If Do you use alcohol or drugs? = Yes, monthly or less<br>Or Do you use alcohol or drugs? = Yes, 2-4 times a month<br>Or Do you use alcohol or drugs? = Yes, 2-3 times a week or more<br><br>Has your life been made worse by your use of alcohol or drugs? |

**Table S5: Coding framework for open-ended gender and sexuality questions.**

This coding framework was developed in partnership with the project's Youth Advisory Group.

| Variable | Scoring |
|---|---|
| Gender* | If participant responds "woman", "female", "girl", "sistergirl", "MTF", "cis female", "cis woman", "trans woman", "trans femme", "female (ish)" or any misspellings of these options, code as 'woman'.<br><br>If participant responds "man", "male", "boy", "brotherboy", "FTM", "cis male", "cis man", "trans man", "trans masculine", "male (ish)" or any misspelling of these options, code as 'man'. If participants response with any of the above first and "and/or non-binary", code as 'man'.<br><br>If participants respond with "non-binary", "enby", "genderfluid", "genderflux", "genderqueer", "agender", "demiboy", "demigirl" or any misspelling of these options, code as 'non-binary'. If participants respond with any of the above first and "and/or man" or "and/or woman", code as 'non-binary. If participants respond with any of the above and "male" or "female" or "AFAB" or "AMAB" in any order, code as 'non-binary'. If participants respond any of the above and "trans femme" or "trans masculine" in any order, code as 'non-binary'.<br><br>If participants respond that they are unsure or questioning their gender, code as 'questioning' (e.g. "idk I'm still figuring it out?" or "???"). If participants respond first with any 'man', 'woman' or 'non-binary' response above followed by questioning (e.g. "woman I think but idk") code as 'man', 'woman' or 'non-binary'.<br><br>If participants respond "prefer not to say" code as 'other'. If participants response with a specific gender identity that cannot be coded as above (e.g. "two-spirit") code as 'other'. If participants provide unusual responses without clarifying context (e.g. "man/woman") code as 'other'. If participants respond using pronouns (e.g. "she/her", "he/they") code as 'other'.<br><br>'Questioning' 'other' are combined with 'non-binary' are combined for analysis due to small cell sizes. 'Man' is used as the reference category for regression analyses.<br><br>Numerical codes:<br>Woman: 0 = man, non-binary/questioning/other; 1 = woman.<br>NBO = 0 = man, woman; 1 = non-binary/questioning/other<br>Man = 0 = non-binary/questioning/other, woman; 1 = man. |
| Gender Diversity* | If participant responds 'yes' to the question "Is your gender different to the gender presumed for you at birth (i.e. the one on your original birth certificate)?", code as 'trans'.<br><br>If participant responds 'no' to the question "Is your gender different to the gender presumed for you at birth (i.e. the one on your original birth certificate)?", code as 'cis'.<br><br>If participant responds 'unsure' to the question "Is your gender different to the gender presumed for you at birth (i.e. the one on your original birth certificate)?", code as 'unsure'.<br><br>If participant responds 'prefer not to say' to the question "Is your gender different to the gender presumed for you at birth (i.e. the one on your original birth certificate)?", code as 'prefer not to say'.<br><br>'Unsure' and 'prefer not to say' are combined for analysis due to small cell sizes. Cisgender is used as the reference category for all regression analyses.<br><br>Numerical codes:<br>Trans: 0 = cis, unsure/prefer not to say; 1 = trans.<br>Cis: 0 = unsure/prefer not to say, trans; 1 = cis.<br>Unsure/prefer not to say: 0 = cis, trans; 1 = unsure/prefer not to say. |
| Sexuality* | Code by sexuality, not romantic attraction (e.g. "panromantic demisexual" codes as 'demisexual'). If participants only respond with romantic attraction, code to nearest sexuality (e.g. "biromantic" codes as 'bisexual/pansexual/omnisexual'). |

| | |
|---|---|
| | If participant responded with more than one sexuality but described one as more accurate or important to them, code as that sexuality.<br><br>If participant responded both ‘gay’ and an identity on the asexuality spectrum (e.g. “gay asexual”), code as ‘asexual’.<br><br>If participant responded any other allosexual identity and an identity on the asexuality spectrum (e.g. “pansexual and asexual”), code as ‘queer’.<br><br>If participants respond that they are unsure or questioning their sexuality, code as ‘questioning’ (e.g. “I have no idea lol”).<br><br>If participant responded any two or more sexualities that do not meet any of the above rules, code as ‘queer’.<br><br>If participants respond “homosexual”, “gay (ish)”, “sapphic”, “mlm”, “wlw” code as ‘gay/lesbian’.<br><br>If participants respond “bi”, “pan”, “omni” code as ‘bisexual/pansexual/omnisexual’.<br><br>If participants respond “ace”, “aroace”, “asexual spec”, “demisexual” code as ‘asexual’.<br><br>If participants respond with “straight” code as ‘other’. If participants respond with a specific sexuality that cannot be coded as above (e.g. “abrosexual”) code as ‘other’. If participants respond that they don’t use any specific identity term (e.g. “I don’t use labels”) code as ‘other’. If participants provide genders they are attracted to without clarifying context (e.g. “I like men”) code as ‘other’.<br><br>‘Questioning’ and ‘other’ are combined with asexual for analysis due to small cell sizes. ‘Gay/lesbian’ is the reference category for all regression analyses.<br><br>Numerical codes:<br>Gay/lesbian: 0 = Bisexual/pansexual/omnisexual, asexual/queer/questioning/other; 1 = gay/lesbian.<br>Bisexual/pansexual/omnisexual: 0 = gay/lesbian, asexual/queer/questioning/other; 1 = Bisexual/pansexual/omnisexual.<br>Asexual/queer/questioning/other: 0 = gay/lesbian, disexual/pansexual/omnisexual; 1 = asexual/queer/questioning/other. |

**Table S6. Pearson correlations between demographics and exposure variables.**

Variable inclusion/exclusion criteria based on deviations from pre-registration (S3). Variables with high correlations with other exposure variables (r >=.70) will be excluded from the analysis (except dummy-coded variables for demographic variables).

| | **1** | **2** | **3** | **4** | **5** | **6** | **7** | **8** | **9** | **10** | **11** | **12** | **13** | **14** | **15** | **16** |
|---|---|---|---|---|---|---|---|---|---|---|---|---|---|---|---|---|
| 1. Age | 1 | -- | -- | -- | -- | -- | -- | -- | -- | -- | -- | -- | -- | -- | -- | -- |
| 2. Disability | .182** | 1 | -- | -- | -- | -- | -- | -- | -- | -- | -- | -- | -- | -- | -- | -- |
| 3. Man (vs else) | .111* | .030 | 1 | -- | -- | -- | -- | -- | -- | -- | -- | -- | -- | -- | -- | -- |
| 4. Woman (vs else) | -.047 | -.177** | -.384** | 1 | -- | -- | -- | -- | -- | -- | -- | -- | -- | -- | -- | -- |
| 5. Non-binary/another (vs else) | -.037 | .156** | -.368** | -.717** | 1 | -- | -- | -- | ---- | -- | -- | -- | -- | -- | -- | -- |
| 6. Cisgender (vs else) | .039 | -.174 | -.080* | .705** | -.649** | 1 | -- | -- | -- | -- | -- | -- | -- | -- | -- | -- |
| 7. Trans (vs else) | -.021 | .163** | .154** | -.655** | .543** | -.843** | 1 | -- | -- | -- | -- | -- | -- | -- | -- | -- |
| 8. Unsure (vs else) | -.032 | .021 | -.134** | -.089* | .191** | -.277** | .543** | 1 | -- | -- | -- | -- | -- | -- | -- | -- |
| 9. Gay/lesbian (vs else) | .034 | -.006 | .150** | -.064 | -.049 | .036 | -.054 | .032 | 1 | -- | -- | -- | -- | -- | -- | -- |
| 10. Bisexual/pansexual/omnisexual (vs else) | -.067 | -.035 | -.099* | .134* | -.060 | .097* | -.091* | -.01 | -.590** | 1 | -- | -- | -- | -- | -- | -- |
| 11. Ace/queer/another sexuality (vs else) | .046 | .048 | -.029 | -.096* | .119* | -.151** | .162** | -.02 | -.277** | -.613** | 1 | -- | -- | -- | -- | -- |
| 12. Fair media representation | -.006 | -.039 | .071 | 0.04 | -.097* | .083* | -.097* | .025 | -.051 | .066 | -.028 | 1 | -- | -- | -- | -- |
| 13. Aboriginal and Torres Strait Islander queerphobia | .155** | .041 | -.015 | -.109* | .121* | -.163** | .161** | .004 | .093 | -.118* | .046 | -.152** | 1 | -- | -- | -- |
| 14. LGBTQA+ racism | .233** | .154** | 0.00 | 0.00 | 0.00 | -0.033 | 0.03 | .005 | .054 | -.099* | .063 | -.224** | .362** | 1 | -- | -- |
| 15. Relationship racism | .126* | .090 | .065 | 0.004 | -.051 | -0.05 | .028 | .038 | -.005 | .034 | -.035 | -.032 | .271** | .381** | 1 | -- |
| 16. Acceptance of LGBTQA+ identity | .137** | -.023 | .025 | -0.023 | .004 | .035 | -.028 | -.012 | .121** | -.095* | -.007 | .001 | -.166** | -.045 | -.130** | 1 |

Note: N = 217-617. **Correlation is significant at the 0.05 level (two-tailed). **correlation is significant at the 0.01 level (two-tailed).*

## Table S7. Pearson correlations between exposure variables and wellbeing outcomes.

Variable inclusion/exclusion criteria based on deviations from pre-registration (S3). Variables with high correlations with other exposure variables (r >=.70) will be excluded from the analysis (except dummy-coded variables for demographic variables).

| | 1 | 2 | 3 | 4 | 5 | 6 | 7 | 8 | 9 | 10 | 11 | 12 | 13 | 14 | 15 | 16 | 17 | 18 | 19 | 20 |
|---|---|---|---|---|---|---|---|---|---|---|---|---|---|---|---|---|---|---|---|---|
| 1. Fair media representation | 1 | -- | -- | -- | -- | ---- | -- | -- | -- | -- | -- | -- | -- | -- | -- | -- | -- | -- | -- | -- |
| 2. Aboriginal and Torres Strait Islander queerphobia | -.152** | 1 | -- | -- | -- | -- | -- | -- | -- | -- | -- | -- | -- | -- | -- | -- | -- | -- | -- | -- |
| 3. LGBTQA+ racism | -.224** | .362** | 1 | -- | -- | -- | -- | -- | -- | -- | -- | -- | -- | -- | -- | -- | -- | -- | -- | -- |
| 4. Relationship racism | -.032 | .271** | .381** | 1 | -- | -- | -- | -- | -- | -- | -- | -- | -- | -- | -- | -- | -- | -- | -- | -- |
| 5 Acceptance of LGBTQA+ identity | -.095* | -.166** | -.045 | | 1 | -- | -- | -- | -- | -- | -- | -- | -- | -- | -- | -- | -- | -- | -- | -- |
| 6. Inner peace | -.059 | -.051 | .050 | -.074 | .323** | 1 | -- | -- | -- | -- | -- | -- | -- | | | -- | | | | |
| 7. Pride LGBTQA+ | -.007 | -.014 | -.144 | -0.07 | .230** | .108* | 1 | -- | -- | -- | -- | -- | | | | | | | | |
| 8. Pride Aboriginal and Torres Strait Islander | -.110* | .070 | .248** | .019 | .129** | .160** | .249** | 1 | -- | -- | -- | -- | -- | -- | -- | -- | -- | -- | -- | -- |
| 9. Family & Kinship | -.021 | -.130* | 0.018 | -.040 | .324** | .325** | .096 | .213** | 1 | -- | -- | -- | -- | -- | -- | -- | -- | -- | -- | -- |
| 10. Culture | -.048 | .038 | .343** | .253** | .077 | .241** | -.012 | .379** | .170** | 1 | -- | -- | -- | -- | -- | -- | -- | -- | -- | -- |
| 11. Spirit | -.055 | -.002 | .262** | .159** | .191** | .412** | .090 | .449** | .278** | | 1 | -- | -- | -- | -- | -- | -- | -- | -- | -- |
| 12. Land & waters | -.059 | .034 | .275** | .146** | .100* | .200** | .018 | .415** | .243** | .432** | .611** | 1 | -- | -- | -- | -- | -- | -- | -- | -- |
| 13. Belonging LGBTQA+ community | -.020 | .033 | -.065 | .042 | .115** | 0.063 | .433** | .019 | .013 | -.058 | .029 | .001 | 1 | -- | -- | -- | -- | -- | -- | -- |
| 14. Belonging Aboriginal & Torres Strait Islander community | .003 | -.129** | .179** | .152** | .156** | .236** | .144* | .404** | .247** | .463** | .530** | .503** | .198** | 1 | -- | -- | -- | -- | -- | -- |
| 15. Belonging Aboriginal & Torres Strait Islander and LGBTQA+ community | .119** | -.122* | .068 | .171** | .146** | 0.08 | .229** | .259** | .163** | .258** | .279** | .248** | .362** | .544** | 1 | -- | -- | -- | -- | -- |
| 16. Participation in LGBTQA+ events | -.058 | .114* | .039 | .113* | .154** | .079 | .352** | .036 | .052 | .077 | .107* | .053 | .366** | .085* | .149** | 1 | -- | -- | -- | -- |
| 17. Participation in Aboriginal and Torres Strait Islander events | -.031 | -.065 | .258** | .148** | .138** | .226** | .029 | .370** | .226* | .570** | .491* | .379** | -.002 | .474** | .216** | .171** | 1 | -- | -- | -- |
| 18. Participation in Aboriginal and Torres Strait Islander LGBTQA+ events | .068 | -.046 | .112* | .147** | .129** | .133** | .130** | .156** | .155** | .362** | .295** | .200** | .137** | .307** | .449** | .360** | .364** | 1 | -- | -- |
| 19. Physical health | -.030 | -.015 | .046 | 0.052 | .142** | .459** | -.053 | .046 | .153** | .139** | .183** | .079 | .005 | .114* | .056 | .056 | .153** | .064 | 1 | -- |
| 20. Substance use impact | -.018 | .091 | .210** | .080 | -.084 | 0.015 | .062 | .060 | -.067 | .074 | .012 | .060 | -.048 | -.093 | -.04 | -.04 | .001 | .136** | -.050 | 1 |

Note: N = 217-617. **Correlation is significant at the 0.05 level (two-tailed). **correlation is significant at the 0.01 level (two-tailed).*

## Table S8. Pearson correlations between demographics and exposure variables

Variable inclusion/exclusion criteria based on deviations from pre-registration (S3). Variables with high correlations with other exposure variables (r >=.70) will be excluded from the analysis (except dummy-coded variables for demographic variables).

| | Age | Disability | Man (vs else) | Woman (vs else) | Non-binary/another (vs else) | Cisgender (vs else) | Trans (vs else) | Unsure (vs else) | Gay/Lesbian (vs else) | Bisexual/pansexual/omnisexual (vs else) | Ace/queer/another sexuality (vs else) |
|---|---|---|---|---|---|---|---|---|---|---|---|
| Inner peace | .287** | -.141** | .029 | .132** | -.154** | .159** | -.147** | -.020 | .083 | -.054 | -.019 |
| Pride LGBTQA+ | .068 | .111* | .009 | -.176** | .171** | -.149** | .161** | -.021 | .033 | -.046 | .021 |
| Pride Aboriginal and Torres Strait Islander | .141** | -.055 | .037 | .158** | -.133** | .094 | -.047 | -.082 | .073 | -.017 | -.053 |
| Family & Kinship | .043 | -.092* | -.045 | .182** | -.149** | .158** | -.164** | .010 | .062 | .021 | -.085* |
| Culture | .084 | -.039 | -.058 | .186** | -.143** | .136** | -.130** | -.010 | -.005 | .092* | -.103* |
| Spirit | .237** | -.063 | -.063 | .138** | -.092* | .111* | -.056 | -.095* | -.003 | .020 | -.020 |
| Land & waters | .100* | -.046 | -.026 | .069 | -.050 | .049 | -.008 | -.072 | -.035 | .061 | -.039 |
| Belonging LGBTQA+ community | -.139** | .032 | .001 | -.186** | .185** | -.197** | .199** | -.006 | .021 | .029 | -.055 |
| Belonging Aboriginal & Torres Strait Islander community | .059 | -.102* | .003 | .082 | -.084 | .051 | -.018 | -.057 | -.005 | .060 | 0.067 |
| Belonging Aboriginal & Torres Strait Islander and LGBTQA+ community | -.048 | -.019 | .062 | -.047 | .001 | -.041 | .049 | -.016 | -.059 | .099* | -.060 |
| Participation in LGBTQA+ events | .056 | .096* | -.011 | -.190** | .199** | -.185** | .212** | -.049 | -.013 | .014 | -.004 |
| Participation in Aboriginal and Torres Strait Islander events | .072 | -.142** | -.145** | .221** | -.113* | .183** | -.156** | -.046 | -.026 | .075 | -.063 |
| Participation in Aboriginal and Torres Strait Islander -LGBTQA+ events | .074 | .061 | -.044 | .045 | -.013 | -.053 | .052 | .002 | -.058 | .124** | -.090* |
| Physical health | -.007 | -.214** | -.014 | .141** | -.131** | .126** | -.113* | -.022 | -.036 | -.039 | .011 |
| Substance use impact | .168** | .095 | .041 | .028 | -.057 | .025 | .010 | -.067 | -.011 | -.055 | .076 |

Note: N = 217-617. **Correlation is significant at the 0.05 level (two-tailed). **correlation is significant at the 0.01 level (two-tailed).*

## Supplementary References:

1. van 't Veer, Anna Elisabeth, and Roger Giner-Sorolla. "Pre-Registration in Social Psychology—a Discussion and Suggested Template." Journal of Experimental Social Psychology 67 (2016): 2-12. https://doi.org/10.1016/j.jesp.2016.03.004.
2. Huria T, Palmer SC, Pitama S, et al. Consolidated criteria for strengthening reporting of health research involving indigenous peoples: the CONSIDER statement. BMC Medical Research Methodology. 2019;19(1):173.

3. Willroth EC, Atherton OE. Best Laid Plans: A Guide to Reporting Preregistration Deviations. *Advances in Methods and Practices in Psychological Science*. 2024;7(1). doi:10.1177/25152459231213802
4. Balsam, Kimberly F., Yamile Molina, Blair Beadnell, Jane Simoni, and Karina Walters. "Measuring multiple minority stress: the LGBT People of Color Microaggressions Scale." *Cultural diversity & ethnic minority psychology* 17, no. 2 (2011): 163.
